# Integrative transcriptomic and metabolomic analysis reveals the molecular basis of leaf variegation in *Cymbidium ensifolium*

**DOI:** 10.3389/fpls.2026.1712811

**Published:** 2026-02-12

**Authors:** Yu Jiang, Jin Wang, Xuliang Tu, Min Zhong, Bin Wan

**Affiliations:** Horticulture Research Institute, Sichuan Academy of Agricultural Sciences, Chengdu, China

**Keywords:** flavonoid biosynthesis, Jianlan, leaf variegation, metabolomics, TF, transcription factor, transcriptomics

## Abstract

**Introduction:**

Leaf variegation is a key ornamental trait of *Cymbidium ensifolium* (Jianlan); however, the molecular mechanisms underlying leaf color mutations in variegated sectors remain poorly understood. Elucidating the regulatory networks associated with pigment variation is essential for both basic research and ornamental improvement.

**Methods:**

Wild-type plants and two leaf color mutants—spot variegation type (BanYi) and line variegation type (XianYi)—were analyzed. Samples were divided into five groups based on leaf origin and color: CK (wild type), B (yellow sectors of BanYi), BL (green sectors of BanYi), X (yellow sectors of XianYi), and XL (green sectors of XianYi). Integrated transcriptomic and metabolomic analyses were performed. Differential expression analysis was conducted across four comparison groups (B vs BL, B vs CK, X vs CK, and X vs XL). Co-expression analysis, metabolite profiling, correlation analysis, and weighted gene co-expression network analysis (WGCNA) were used to identify key regulatory genes and modules associated with pigment accumulation.

**Results:**

A total of 6,716 differentially expressed genes (DEGs) were identified, with 141 shared among all comparison groups. These DEGs were significantly enriched in phenylpropanoid biosynthesis, zeatin biosynthesis, and flavonoid biosynthesis pathways. Twenty-four DEGs involved in flavonoid biosynthesis, including structural genes such as *CCOAMT*, *TT4*, and *HCT*, showed elevated expression in variegated leaf sectors. In addition, 80 transcription factors from the MYB, bHLH, and WRKY families were co-expressed with 50 pigment-related DEGs, suggesting coordinated transcriptional regulation. Metabolomic analysis identified 3,024 differentially accumulated metabolites (DAMs), of which 56 were shared across all groups. Correlation analysis revealed strong associations between co-DEGs and co-DAMs. WGCNA further identified key modules, including the “MEtan” module, which contained 83 genes significantly correlated with pigment-related metabolites such as 1′-Hydroxy-γ-carotene and violaxanthin.

**Discussion:**

These results demonstrate that leaf variegation in *C. ensifolium* is regulated by coordinated transcriptional and metabolic networks, particularly involving flavonoid and carotenoid-related pathways. The identified structural genes, transcription factors, and co-expression modules provide novel insights into the genetic basis of leaf color variation and offer valuable candidate targets for the ornamental improvement of Jianlan.

## 1 Introduction

The Orchidaceae is one of the largest families of angiosperms, comprising over 25, 000 species (Christenhusz and Byng, 2016). Among them, *Cymbidium* is a highly valued genus with significant ornamental and economic importance. *Cymbidium ensifolium* (Jianlan), known for its diverse flower colors, pleasant fragrance, and varied leaf morphology, has long been favored by both breeders and consumers. Through traditional hybridization and mutagenesis breeding, numerous cultivars with various flower forms, leaf colors, and aromas have been developed (Zhu GenFa et al., 2004; Li et al., 2023; Jiang et al., 2024). While flower color and fragrance have traditionally been the main focus in orchid breeding, leaf color variation has recently gained increasing attention. In *C. ensifolium*, a distinct leaf color variation known as “leaf variegation”, characterized by golden stripes or spots on the leaves, presents high ornamental value. However, compared to floral traits, the molecular mechanisms underlying leaf color variation remain poorly understood, partly due to the more complex regulatory pathways involved (Gao et al., 2020). Therefore, elucidating the molecular basis of leaf color mutation in *C. ensifolium* is not only essential for understanding its regulatory mechanisms but also holds great potential for the breeding of ornamental leaf-color varieties.

The coloration of plant leaves is mainly determined by three types of pigments: chlorophylls, carotenoids, and flavonoids (Iwashina, 2015; Zhao et al., 2016). Among them, flavonoids are a group of important phenylpropanoid-derived secondary metabolites, widely studied due to their roles in pigmentation, antioxidation, and stress resistance (Liu et al., 2021a). Based on their structural features, flavonoids can be categorized into 12 subclasses, including anthocyanins, flavonols, chalcones, stilbenes, and others (Winkel-Shirley, 2001; Sasaki and Nakayama, 2015). The biosynthesis of flavonoids begins with the formation of 4-coumaroyl-CoA via the phenylpropanoid biosynthesis pathway. Its key intermediate, dihydroflavonol, is primarily synthesized through the catalytic actions of *CHS*, *CHI*, *F3H*, and two cytochrome P450 enzymes (*F3’H* or *F3’5’H*) (Saito et al., 2013; Sun et al., 2015; Nabavi et al., 2020). Dihydroflavonol can then be converted into kaempferol, quercetin, and myricetin by *flavonol synthase* (*FLS*) (Meng et al., 2019), or into anthocyanins and proanthocyanidins via the action of *dihydroflavonol 4-reductase* (*DFR*) (Meyer et al., 1987; LaFountain and Yuan, 2021), resulting in distinct color phenotypes in plants.

Previous studies have shown that leaf color variation in Cymbidium species is closely associated with the composition of flavonoid compounds (Gao et al., 2020; Jiang et al., 2024; Mei et al., 2024). The biosynthesis of flavonoids is not only regulated by structural genes but also by multiple transcription factors (TFs) that act in a coordinated manner. Studies have shown that MYB, bHLH, and WD40 transcription factors form the classic MBW complex, playing a central role in the regulation of flavonoid biosynthesis, especially anthocyanin production (Bulanov et al., 2025). In addition, other TF families such as *NAC*, *WRKY*, *ERF*, and *HY5* have also been implicated in the transcriptional regulation of flavonoid metabolism across various plant species (Zhang et al., 2020; Liu et al., 2021b). These TFs have been reported to play crucial roles in pigment accumulation in diverse horticultural plants, including grape (Jin et al., 2025), Arabidopsis (Morishita et al., 2009), apple (Wang et al., 2024), and orchid species (Yang et al., 2025). Therefore, identifying flavonoid-related TFs and their regulatory networks is essential for dissecting the molecular mechanisms of leaf color variation in *C. ensifolium*.

In recent years, the integration of transcriptomic and metabolomic analyses has emerged as a powerful approach for investigating the molecular mechanisms underlying plant metabolic regulation, particularly in pigment-related pathways (Zhang et al., 2023; Guan et al., 2024; Yang et al., 2024). For example, Mei et al. applied an integrative analysis strategy and revealed the critical roles of anthocyanin metabolism in leaf color variation in C. ensifolium (Mei et al., 2024). Similar approaches have also been successfully applied to other horticultural species, such as bitter melon, where candidate genes (e.g., *MYB*s, *NAC*s, and *bHLH*s) involved in flavonoid biosynthesis were identified based on color variation (Yang et al., 2024). The recent release of a high-quality, chromosome-scale reference genome for *C. ensifolium* (Ai et al., 2021) has laid a solid foundation for further molecular studies on leaf color mutations.

In this study, we conducted integrated transcriptomic and metabolomic analyses on wild-type *C. ensifolium* and two leaf variegation (spot variegation and line variegation) to investigate gene expression and metabolite accumulation associated with different leaf color phenotypes. We identified key metabolites and candidate genes potentially involved in leaf color variation, as well as co-expressed TFs that may play regulatory roles. We identified the significantly correlated gene modules MEtan and MEpink with violaxanthin and beta-carotene metabolites through WGCNA. Furthermore, we examined the expression profiles of differentially expressed genes (DEGs) in the flavonoid biosynthetic pathways. Our findings provide novel insights into the transcriptional and metabolic basis of leaf color variation in *C. ensifolium* and offer a molecular framework for the breeding of ornamental leaf variegation traits.

## 2 Materials and methods

### 2.1 Plant materials

The wild-type Suhua Jianlan were obtained from the Orchid Greenhouse of the Horticulture Research Institute of the Sichuan Academy of Agricultural Sciences. Wild-type green rhizomes of Jianlan were cultured *in vitro* using MS medium supplemented with a high concentration of 6-benzylaminopurine (6-BA, 2.0–3.0 mg/L). During culture, some rhizomes exhibited somaclonal variation and developed a yellow phenotype. These yellow rhizomes were subsequently induced on MS medium containing 6-BA (1.0–2.0 mg/L) and α-naphthaleneacetic acid (NAA, 0.2 mg/L), which promoted differentiation into plantlets displaying spot and line variegation phenotypes. All cultures were maintained under a 16 h light/8 h dark photoperiod at 25°C during the day and 22°C at night.

To investigate the molecular basis of leaf color variation in variegated Jianlan, leaf tissues were collected from healthy and phenotypically stable plants. For the spot variegation type, both the green sectors (BL) and the yellow sectors (B) were collected. For the line variegation type, both the green sectors (XL) and the yellow sectors (X) were collected. Wild-type leaves with uniform green coloration (CK) were used as controls. For each group, three biological replicates were collected. All samples were immediately frozen in liquid nitrogen after collection and subjected to both transcriptome and metabolome analyses.

### 2.2 RNA extraction and transcriptome sequencing

Total RNA was extracted from leaf samples using the Plant Total RNA Extraction Kit (Invitrogen, USA) following the manufacturer's instructions. RNA purity and integrity were assessed via agarose gel electrophoresis. RNA concentration was measured using the Qubit RNA BR Assay Kit (Q10210), and RNA integrity was evaluated using the Agilent Bioanalyzer 2100 system. For each sample, 5 μg of total RNA was used to construct RNA-seq libraries. Libraries were prepared using the Illumina TruSeq Stranded mRNA Library Prep Kit and sequenced on the Illumina HiSeq 2500 platform. Briefly, Poly-(A) mRNA was isolated from total RNA using Oligo-(dT) magnetic beads and fragmented in fragmentation buffer. First-strand cDNA was synthesized using random hexamer primers, followed by second-strand synthesis using DNA polymerase I, dNTPs, buffer, and RNase H to generate double-stranded (ds) cDNA. The resulting cDNA fragments were purified using AMPure XP beads (Beckman Coulter), targeting a preferred length of 250–300 bp. To ensure library quality, PCR products were further purified with AMPure XP beads and assessed using the Agilent Bioanalyzer 2100 system. Indexed libraries were clustered using the TruSeq PE Cluster Kit v4-cBot-HS (Illumina) on the cBot system and sequenced on the Illumina HiSeq-PE150 platform.

### 2.3 Differential expression analysis

Raw sequencing reads were processed using fastp (version 0.24.1) (Chen, 2023) to remove adapter sequences and low-quality reads. Clean paired-end reads were then aligned to the *C. ensifolium* (Jianlan) reference genome (Ai et al., 2021) using HISAT2 (version 2.2.1) with default parameters. The resulting alignment files were sorted with SAMtools (version 1.19.2) (Li et al., 2009). Gene-level quantification was performed using featureCounts (version 2.0.6) (Liao et al., 2014) with parameters -p -B -C, and expression levels were normalized as fragments per kilobase of transcript per million mapped reads (FPKM).

Differentially expressed genes (DEGs) were identified using DESeq2 (version 1.42.1) (Love et al., 2014) across four comparison groups: B vs BL, B vs CK, X vs CK, and X vs XL. Genes with |log_2_(fold change)| ≥ 1 and a false discovery rate (FDR) ≤ 0.05 in any comparison were considered significantly differentially expressed. Expression heatmaps of DEGs were visualized using the pheatmap package version 1.0.12 (Kolde, 2015).

### 2.4 Identification of TFs

Protein sequences of plant transcription factors were retrieved from PlantTFDB v5.0 (https://planttfdb.gao-lab.org/) (Tian et al., 2020). These sequences were aligned to the Jianlan reference genome using DIAMOND version 2.1.10 blastp (parameters: -e 1e-20 –approx-id 60 –query-cover 60) (Buchfink et al., 2015). Genes in the Jianlan genome with significant hits to known transcription factor proteins were identified as putative transcription factors. Each gene was assigned to a TF family based on its best-hit match in the PlantTFDB database.

### 2.5 Functional enrichment analysis

Gene Ontology (GO) annotations were performed using EggNOG-mapper version 2.0.1 (Cantalapiedra et al., 2021). Kyoto Encyclopedia of Genes and Genomes (KEGG) annotations were conducted using KOBAS version 3.0 (Bu et al., 2021), with *Arabidopsis thaliana* selected as the reference species. All annotated genes in the *C. ensifolium* genome were used as the background gene set, Enrichment analysis of DEGs was carried out using the R package clusterProfiler version 4.10.1 (Wu et al., 2021). GO terms or KEGG pathways with a Benjamini–Hochberg adjusted *p-value* < 0.05 were considered significantly enriched.

### 2.6 Metabolite extraction and identification

Leaf samples of *C. ensifolium* stored at −80°C were placed on ice prior to extraction. A total of 20 μL of sample was mixed with 120 μL of 50% methanol solution (4°C) and homogenized for 1.5 min using a mixer mill equipped with zirconia beads (MM 400, Retsch). The homogenates were incubated at 25°C for 15 min, followed by overnight extraction at −20°C. The extracts were then centrifuged at 4200 × g for 15 min, and the resulting supernatants were filtered and transferred into 96-well plates. Metabolomic profiling of 18 C*. ensifolium* samples, including quality control (QC) samples, was performed using a high-performance liquid chromatography system coupled with a high-resolution tandem mass spectrometer (TripleTOF 6600, Sciex) operated in both positive and negative ionization modes. Raw data were preprocessed using the XCMS software package, which aligned features based on the precursor ion mass-to-charge ratio (m/z) and retention time, and extracted chromatographic peak areas for quantification. Metabolite identification was achieved by matching precursor ion m/z values and corresponding MS/MS fragment ions against standard compounds in public and in-house databases.

### 2.7 Principal component analysis and identification of differentially accumulated metabolites

Gene expression and metabolite quantification matrices were subjected to Z-score normalization. Then, PCA and hierarchical clustering were performed using the prcomp function and the pheatmap package (version 1.0.12) in R, respectively. Partial least squares discriminant analysis (PLS-DA) was conducted using the opls function from the ropls package version 1.42.0 (Thévenot et al., 2015), and variable importance in projection (VIP) values were calculated. Differentially accumulated metabolites (DAMs) were identified based on the following thresholds: VIP ≥ 1, |log_2_(fold change)| ≥ 1, and P-value < 0.05. DAMs were screened across four pairwise comparison groups: B vs BL, B vs CK, X vs CK, and X vs XL.

### 2.8 Correlation analysis

To evaluate the association between DEGs and DAMs, as well as between differentially expressed structural genes and TFs, Pearson correlation coefficients were calculated using the cor function in R (method = "pearson"). Pairs with an absolute correlation coefficient (|r|) ≥ 0.8 and a Q-value < 0.05 were considered significantly correlated. The resulting correlation networks were visualized using Cytoscape version 3.10.3 (Shannon et al., 2003).

### 2.9 Weighted gene co-expression network analysis

WGCNA was performed using the R package WGCNA (version 1.73) (Langfelder and Horvath, 2008). The expression matrix of 24, 373 genes for all 15 samples was used as input file, and the top 25% of genes with the highest variance were selected for network construction. Sample clustering was conducted using the hclust function with the average linkage method (method = "average"). The soft-thresholding power (β) was determined using the pickSoftThreshold function and set to 8 (**Supplementary Figure S1**). Co-expression modules were identified using the blockwiseModules function with the following parameters: TOMType = "unsigned", minModuleSize = 30, and mergeCutHeight = 0.25.

Module eigengenes (MEs) were calculated using the moduleEigengenes function. The Pearson correlation coefficients between MEs and eight pigment-related DAMs, including Zeaxanthin, Violaxanthin, β-Carotene, 1’-Hydroxy-γ-carotene, Naringin, 8, 12-Diethylbacteriochlorophyllide d, Pheophorbide a, and Coproporphyrinogen III, were computed. Modules with a |r| greater than 0.8 and P < 0.05 were considered significantly associated with the phenotypic traits.

### 2.10 Quantitative real-time PCR

Complementary DNA (cDNA) was synthesized from 1 µg of total RNA using the iScript cDNA Synthesis Kit (Bio-Rad, Hercules, CA, USA) to verify RNA-seq results. Quantitative real-time PCR (qRT-PCR) was performed on the 15 samples corresponding to the RNA-seq samples using SYBR Green QPCR Mix (DF Biotech, Chengdu, China) on a CFX384 Real-Time PCR System (Bio-Rad, Hercules, CA, USA). Gene expression levels were normalized using the *EF1a* (elongation factor 1-alpha) as an internal reference gene. Relative expression was calculated using the comparative Ct method. The primer sequences (5’-3’) used for qRT-PCR were: *EF1a* (forward: CTACCAAGCTTCAAAGGATG; reverse: CTCAGATACAGTAGTAGACC), *JL003847* (forward: CGCCGATGTTATTGCTGTGG; reverse: CATAAGCACCGTTCGGCATG), *JL014616* (forward: CGAAGGGCCTCCACTTTCTT; reverse: CGGAAGGACGGAGATAACGG), *JL022919* (forward: AGGATTTGGTGGCTCAGCTC; reverse: CAAACCGGCCGTTCAATAGC), *JL015258* (forward: TACTGGGCGAGGATTGAGGA; reverse: GCGAAACGTGGCCTTTTCTT) and *JL024060* (forward: TCTTGCTTGTCTCGACCACC; reverse: CAGCGGCGTAGAATTTGACG).

## 3 Results

### 3.1 Spot variegation and line variegation

**Figure 1:**
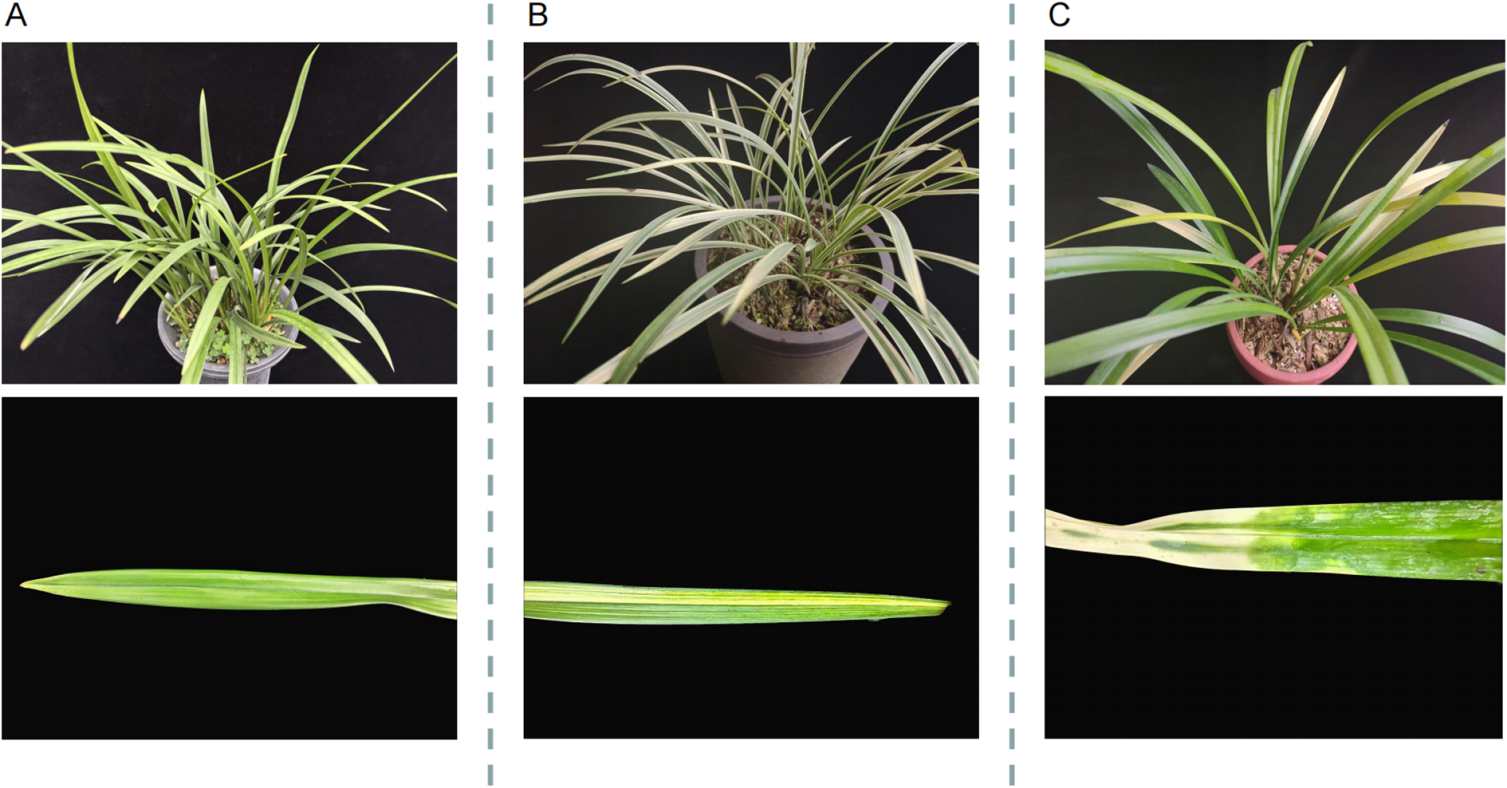
Wild-type (CK) **(A)**, line variegation type (XianYi) **(B)** and spot variegation type (BanYi) **(C)** phenotypes in *C . ensifolium*. The top figure shows the overall plant, and the bottom figure shows details of the plant's leaves.

In this study, two types of leaf variegation, namely line variegation and spot variegation, were obtained through hormone induction. The entire plant of wild-type Jianlan is almost entirely green (**Figure 1A**). The line variegation exhibits longitudinal yellow-white or light-green bands aligned along the leaf veins, extending from the leaf base to the tip (**Figure 1B**). These stripes are relatively regular and consistent among different leaves, generally following the venation pattern. In contrast, spot variegation plants exhibited irregular yellow/white mosaic patches interspersed with green sectors; the patch edges were less regular and often formed sectorial blocks across the blade (**Figure 1C**). These two mutant types provide germplasm resources for Jianlan breeding.

### 3.2 Transcriptomic expression characteristics associated with leaf variegation

**Figure 2:**
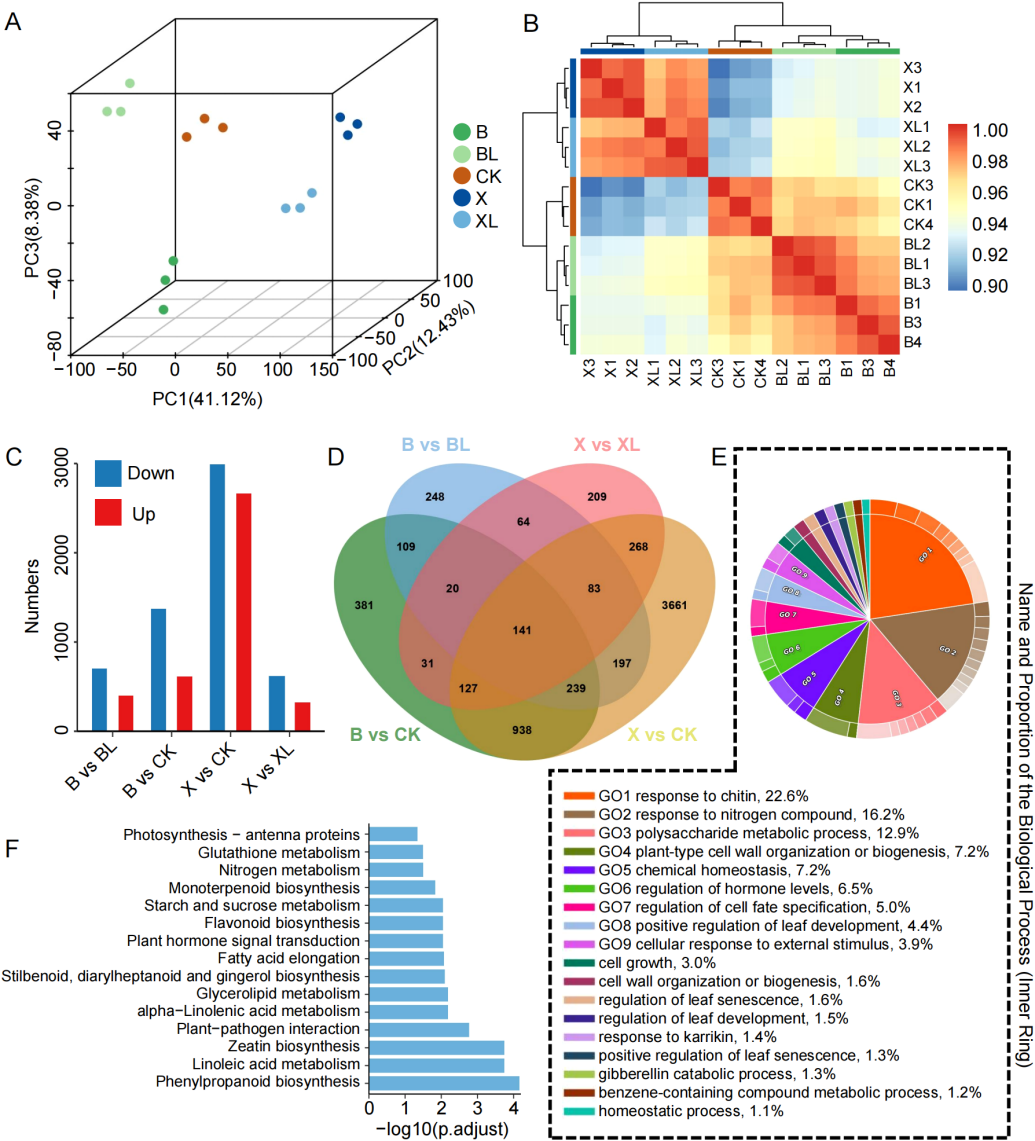
Transcriptomic characteristics of *C . ensifolium* leaves. **(A)** 3D-PCA based on transcriptome data. **(B)** Hierarchical clustering heatmap based on transcriptomic data showing the clustering patterns of the samples. **(C)** Bar chart illustrating the number of upregulated and downregulated DEGs in four comparison groups. **(D)** Venn diagram showing the overlap of DEGs among different comparison groups. **(E)** Circos plot depicting GO enrichment results of the DEGs. **(F)** Bar chart showing KEGG enrichment results of the DEGs.

To elucidate the molecular mechanisms underlying leaf variegation mutation in *C.ensifolium*, we performed Illumina RNA sequencing on CK and two variegated cultivars, spot variegation (B/BL) and line variegation (X/XL), using green (BL and XL) and yellow (B and X) leaf sectors with three biological replicates per sample. Each sample yielded 3.04–3.65 GB of clean data and 20, 277, 516–24, 354, 992 paired-end clean reads. The clean reads were mapped to the *C. ensifolium* reference genome with a high mapping rate of 95.04%–96.19% (**Supplementary Table S1**), indicating that the sequencing data were of high quality and reliability. 3D-PCA revealed that the first two components accounted for 71.43% of the total variance (PC1: 57.77%, PC2: 13.66%, PC3: 8.38%). Samples clustered according to their respective treatment groups, and biological replicates grouped closely together, indicating high intra-group consistency and clear inter-group differences (**Figure 2A**). The high concordance of gene expression profiles among biological replicates was consistent with the PCA results, further confirming the reliability of the transcriptomic data (**Figure 2B**). Differential expression analysis identified 6716 DEGs across four pairwise comparisons (B vs BL, B vs CK, X vs CK, and X vs XL). The greatest transcriptional divergence was observed in the X vs CK comparison, with 2, 664 upregulated and 2, 990 downregulated genes (**Figure 2C**; **Supplementary Figure S2**). In contrast, the fewest DEGs were detected in X vs XL, with only 324 upregulated and 619 downregulated genes. Notably, B vs CK and X vs CK shared the highest number of overlapping DEGs (1, 445 genes). Additionally, 141 DEGs were commonly identified across all four comparisons, which we defined as co-DEGs potentially associated with leaf color variation in *C. ensifolium* (**Figure 2D**).

To further explore the biological significance of these DEGs, we conducted GO and KEGG enrichment analyses using the 6, 716 genes identified across the four comparison groups. GO terms significantly enriched among DEGs included “response to chitin” (22.6%), “response to nitrogen compound” (16.2%), “polysaccharide metabolic process” (12.9%), and “regulation of hormone levels” (6.5%) (**Figure 2E**). KEGG pathway analysis showed that DEGs were predominantly enriched in pathways such as phenylpropanoid biosynthesis, zeatin biosynthesis, plant–pathogen interaction, plant hormone signal transduction, flavonoid biosynthesis, and photosynthesis - antenna proteins (**Figure 2F**; **Supplementary Table S2**). These findings suggest that the yellow leaf phenotype in mutant cultivars may be closely related to phenylpropanoid metabolism, flavonoid biosynthesis, and plant hormone signaling pathways.

### 3.3 Expression of DEGs in the flavonoid biosynthesis pathway

**Figure 3:**
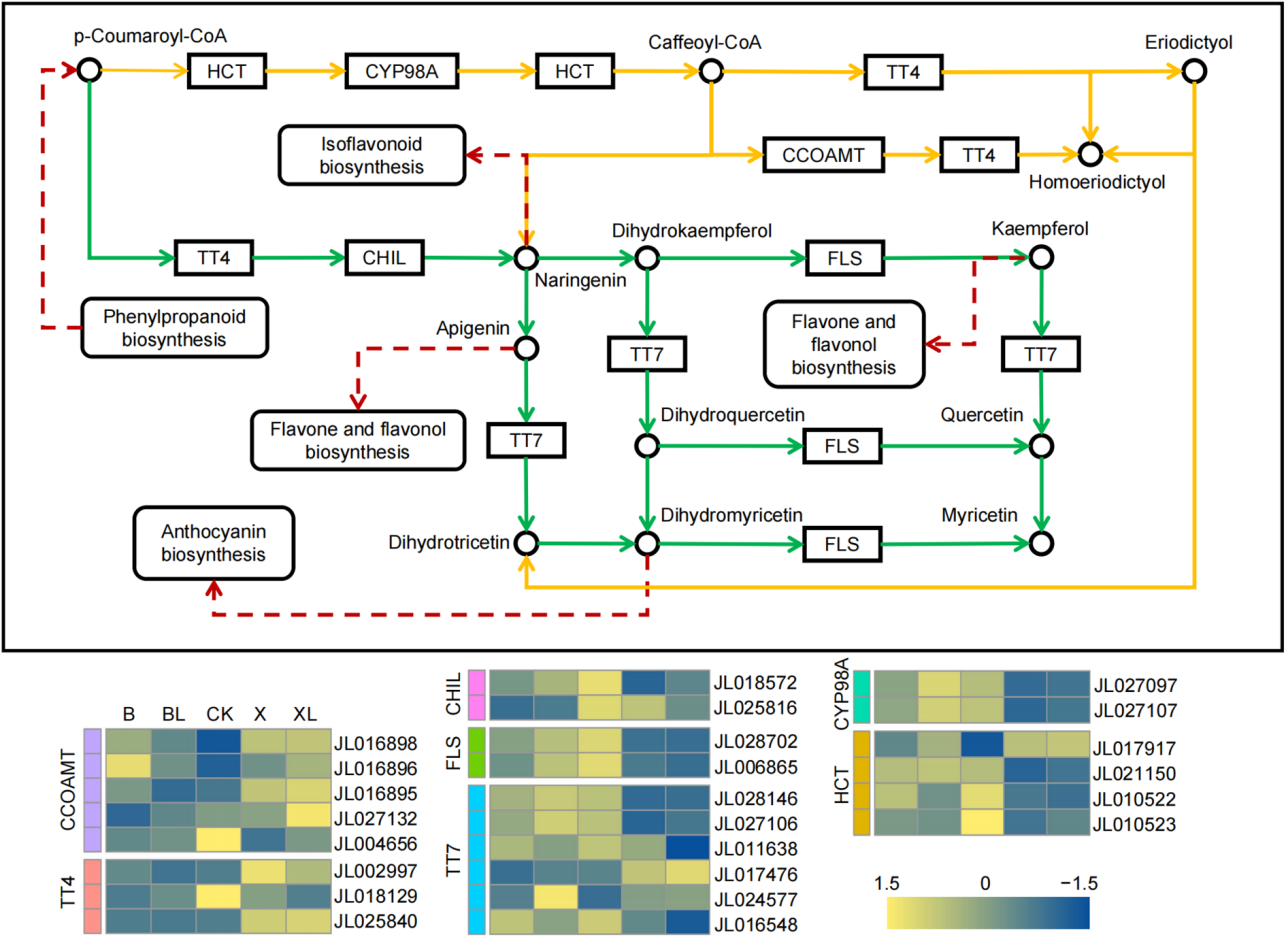
Expression of key DEGs involved in the Flavonoid biosynthesis pathway in *C. ensifolium* leaf tissue. The top is a schematic diagram of the Flavonoid biosynthesis pathway in C. ensifolium; the rectangles indicate gene names, the circular rectangles represent related biosynthetic pathways, and the circles represent specific metabolites. The bottom is a heatmap of different genes expression in the Flavonoid biosynthesis pathway, with expression levels normalized by Z-scores, ranging from -1.5 (low expression) to 1.5 (high expression).

Flavonoids, a major class of plant polyphenolic secondary metabolites, play diverse biological roles, including contributing to pigmentation and exhibiting antioxidant activity. Transcriptome analysis revealed that DEGs were significantly enriched in the flavonoid biosynthesis pathway, suggesting that this pathway is actively regulated under different conditions. A total of 24 DEGs involved in flavonoid biosynthesis pathway (**Figure 3**). These DEGs were annotated as key enzymes in the pathway, including *CCOAMT* (*caffeoyl-CoA 3-O-methyltransferase*), *TT4* (*Chalcone and stilbene synthase*), *CHIL* (*Chalcone-flavanone isomerase-like*), *FLS* (*flavonol synthase*), and *HCT* (*hydroxycinnamoyl-CoA transferase*). Among them, eight DEGs (specifically, one *CCOAMT*, one *TT4*, two *CHIL*, two *FLS*, and two *HCT* genes) exhibited higher expression levels in the CK group, suggesting a relatively active flavonoid biosynthetic process under control conditions. In contrast, one *CCOAMT* gene (*JL016896*) was predominantly expressed in the B group, while two *TT4* genes (*JL002997* and *JL025840*) and one *HCT* gene (*JL017917*) showed markedly higher expression levels in the X group. This expression pattern in the X group may contribute to enhanced accumulation of naringenin, a central intermediate in the flavonoid biosynthesis pathway. The increased availability of naringenin could subsequently promote flux through downstream branches of the pathway, including isoflavonoid biosynthesis and flavone and flavonol biosynthesis, potentially altering the metabolic composition to contribute to the formation of the line variegation phenotype.

### 3.4 TFs involved in leaf color regulation

**Figure 4:**
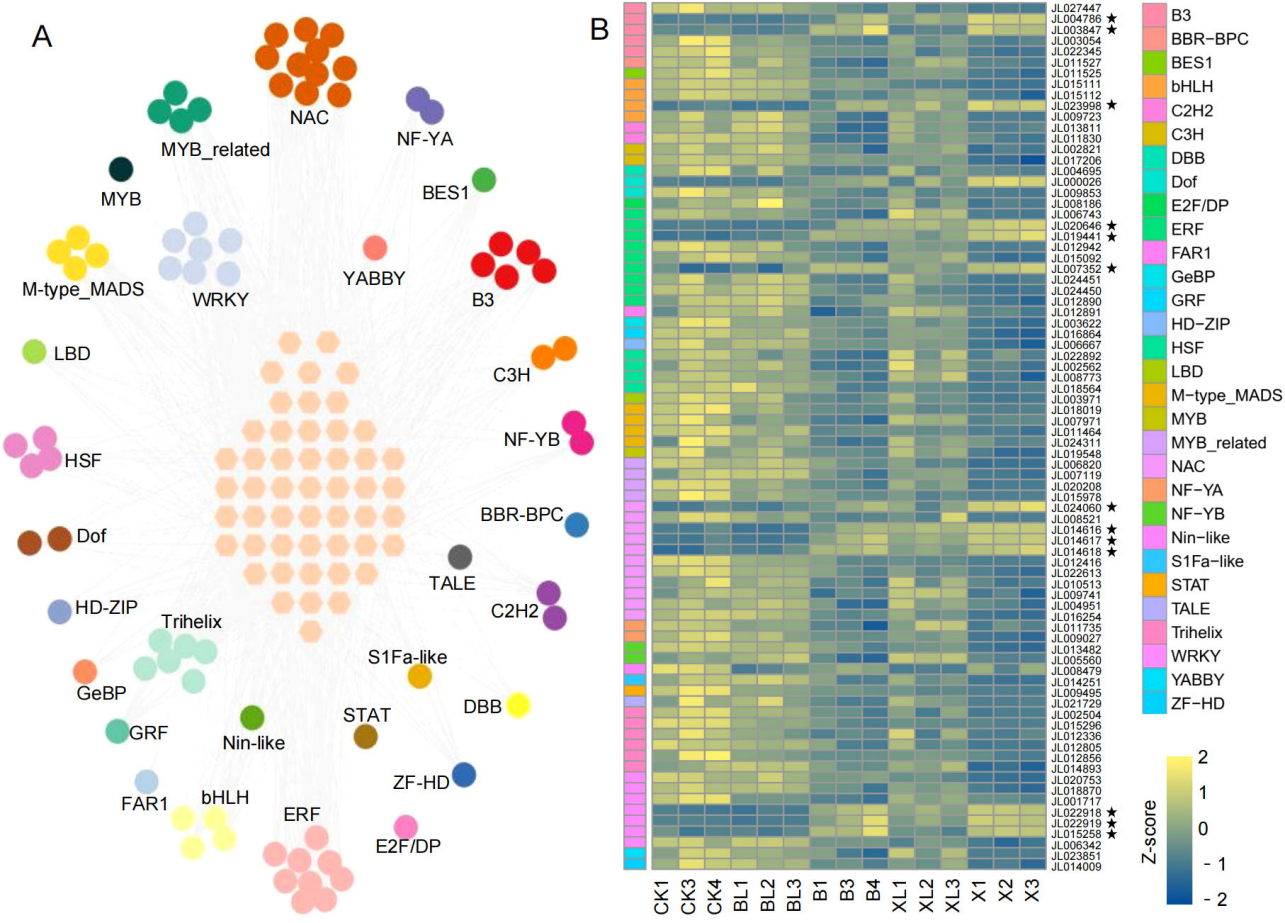
Co-expression analysis between TFs among co-DEGs and other DEGs. **(A)** Co-expression network illustrating the regulatory relationships between TFs identified within the co-DEGs and other DEGs. Pink nodes in the center represent the DEGs. **(B)** Heatmap showing the expression profiles of 80 TFs from the regulatory network across different samples. The genes marked with a pentagram are those highlighted in the main text.

Given the pivotal roles of TFs in regulating gene expression during plant development and physiological processes, we identified TFs in *C. ensifolium*. A total of 9, 404 TFs were identified, among which 2800 showed differential expression across the various comparison groups. Notably, 85 TFs were included in the co-DEGs, with the majority belonging to the *NAC*, *ERF*, and *WRKY* families (**Supplementary Table S3**). To further investigate the regulatory role of these TFs in leaf color variation, we calculated the pearson correlation coefficient (r) between the expression profiles of TFs within the co-DEGs and other functional DEGs. The analysis revealed 912 significantly co-expressed TF-DEG pairs (|r| ≥ 0.8, Q-value < 0.05), involving 80 TFs and 50 DEGs (**Figure 4A**). Among them, members of the *NAC*, *ERF*, *WRKY*, *M-type_MADS*, and *Trihelix* families exhibited extensive co-expression with other genes, with 39, 39, 38, 29, and 27 co-expressed DEGs, respectively. These TFs are likely to play key roles in the transcriptional regulation underlying leaf color variation in *C. ensifolium*. Furthermore, to verify the reliability of the RNA-seq results, we performed qRT-PCR analysis on five differentially expressed TFs (*JL003847*, *JL014616*, *JL024060*, *JL022919*, and *JL015258*). Their expression trends were largely consistent with the transcriptome sequencing results (**Supplementary Figure S3**).

Compared with green leaf samples (CK, XL, BL), several TFs displayed significantly higher expression in the yellow leaf mutants (B and X), including two *B3* TFs (*JL004786*, *JL003847*), one *bHLH* TF (*JL023998*), three *ERF* TFs (*JL020646*, *JL019441*, *JL007352*), four *NAC* TFs (*JL024060*, *JL014616*, *JL014617*, *JL014618*), and three *WRKY* TFs (*JL022918*, *JL022919*, *JL015258*) (**Figure 4B**). In contrast, *MYB* family TFs showed higher expression in green leaf samples compared to yellow ones, suggesting that *MYB* TFs might be associated with the maintenance of green pigmentation, while the upregulated TFs in the yellow mutants may contribute to chlorophyll degradation or altered pigment biosynthesis.

### 3.5 Metabolomic profiling reveals differential accumulation of metabolites associated with leaf color variation in *C. ensifolium*

**Figure 5:**
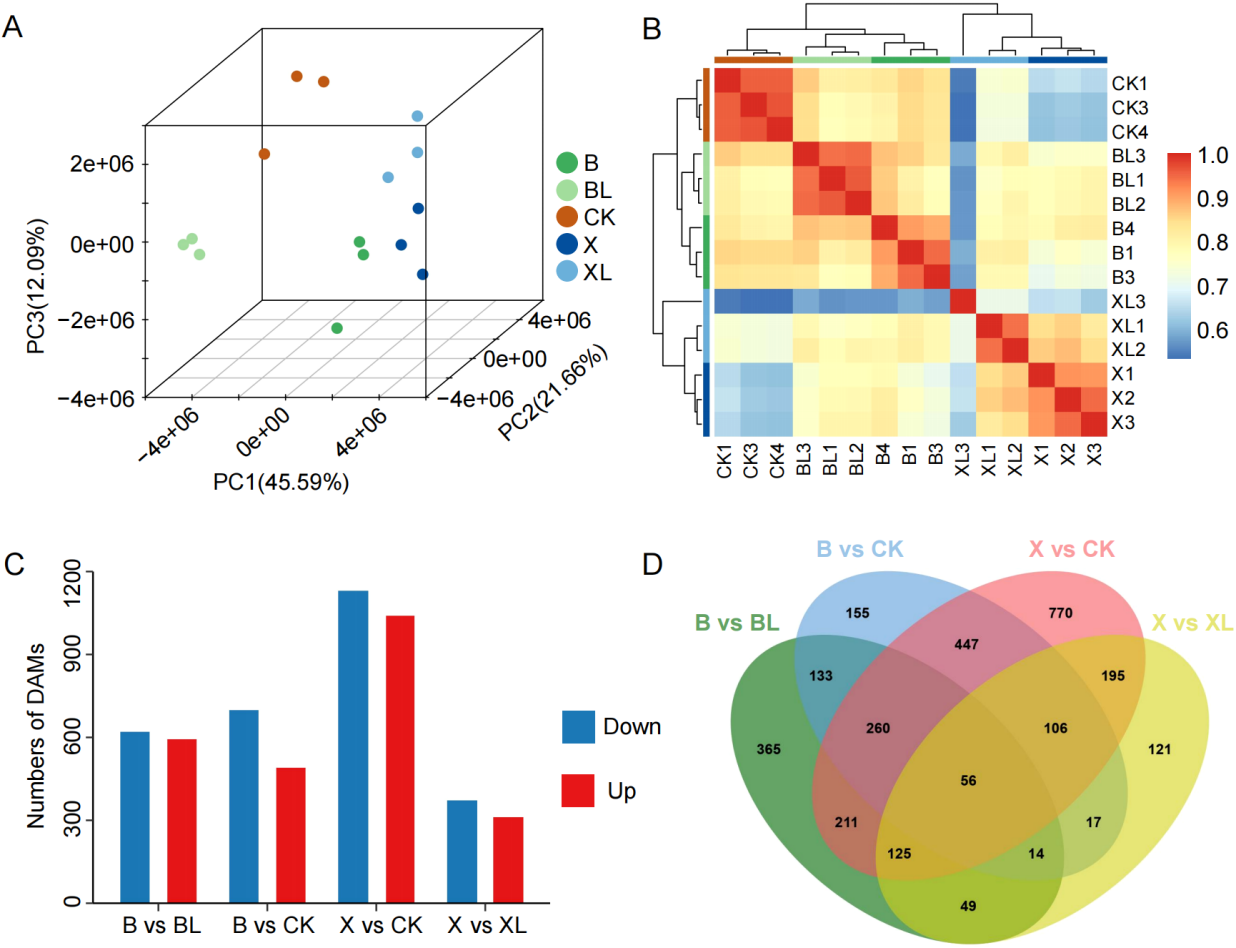
Metabolomic profiling of *C . ensifolium* leaves. **(A)** 3D-PCA based on metabolomic data from samples, with different colors representing five groups. **(B)** Correlation heatmap showing hierarchical clustering of the samples. **(C)** Bar plot showing the numbers of upregulated and downregulated DAMs in four comparison groups. **(D)** Venn diagram illustrating the overlap of DAMs among different comparison groups.

To investigate the metabolic basis underlying leaf color variation in *C. ensifolium*, we performed untargeted metabolomic profiling on green and yellow leaves from CK, spot variegation (BL and B), and line variegation (XL and X). A total of 5, 252 metabolites were identified across all samples (**Supplementary Table S4**). 3D-PCA and correlation heatmaps showed clear separation among the five sample groups and high reproducibility among biological replicates, indicating distinct metabolic profiles (**Figures 5A, B**). DAMs were identified using thresholds of VIP ≥ 1, |log2(fold change)| ≥ 1, and P < 0.05. Four pairwise comparisons were conducted: B vs BL, B vs CK, X vs CK, and X vs XL. The number of DAMs varied widely among comparisons, with the X vs CK group showing the highest number (2, 170 DAMs; **Figure 5C**). Specifically, compared to the yellow leaves of spot variegation (B), 593 and 490 upregulated DAMs and 620 and 698 downregulated DAMs were detected in BL and CK, respectively. Similarly, compared to the yellow leaves of line variegation (X), 1, 040 and 311 upregulated DAMs and 1, 130 and 372 downregulated DAMs were found in XL and CK, respectively. Importantly, 56 DAMs were shared across all four comparisons (**Figure 5D**). These were defined as co-DAMs and are likely key metabolites involved in the formation of yellow leaf coloration in *C. ensifolium*.

### 3.6 Regulatory associations between DEGs and DAMs in leaf variegation

**Figure 6:**
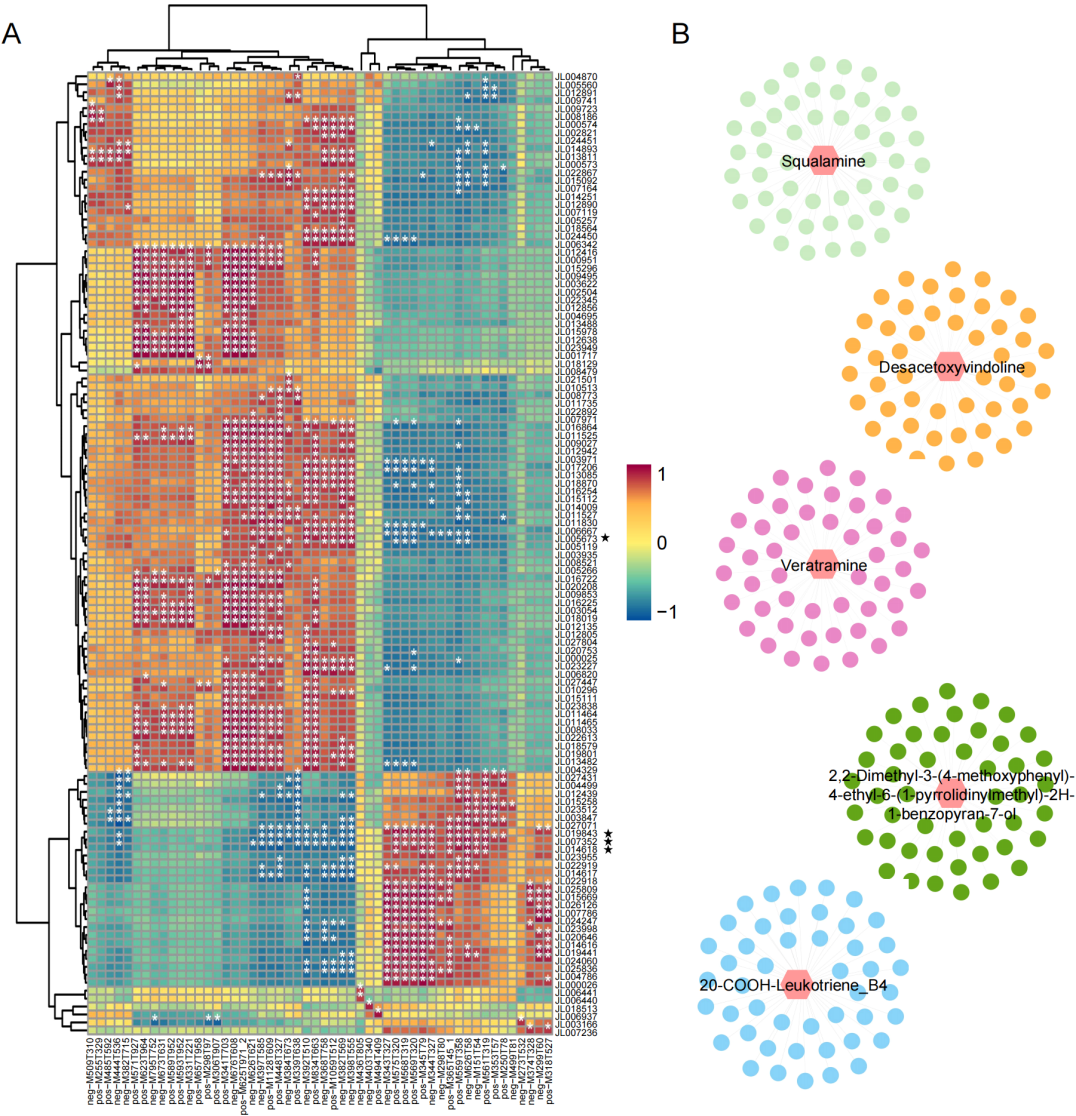
Correlation analysis between co-DEGs and co-DAMs. **(A)** Heatmap showing the correlation coefficients between DEGs and DAMs. * indicate strong correlations with |r| ≥ 0.8. The genes marked with a pentagram are those highlighted in the main text. **(B)** Gene–metabolite regulatory network of the top five DAMs that are most strongly regulated by DEGs.

To investigate the relationship between DAMs and DEGs involved in the leaf variegation of *C. ensifolium*, we performed a correlation analysis between 56 co-DAMs and 141 co-DEGs. A total of 1, 098 significant DEG–DAM pairs were identified, involving 121 DEGs and 52 DAMs. Among them, 904 pairs were positively correlated and 194 were negatively correlated (**Figure 6A**; **Supplementary Table S5**). Notably, *JL007352*, *JL014618*, *JL019843*, and *JL005673* showed significant correlations with a larger number of metabolites, being associated with 23, 22, 21, and 20 metabolites, respectively. In addition, we constructed a correlation regulatory network (**Supplementary Figure S4**), which highlighted key gene–metabolite interactions. Among them, neg-M626T621, neg-M397T585, pos-M448T327, neg-M392T510, and neg-M382T569 were found to be regulated by multiple genes (**Figure 6B**). These results provide important insights into the molecular mechanisms underlying leaf color variation in *C. ensifolium*.

### 3.7 Gene co-expression modules associated with pigment-related metabolites in *C. ensifolium* leaf color mutants

**Figure 7:**
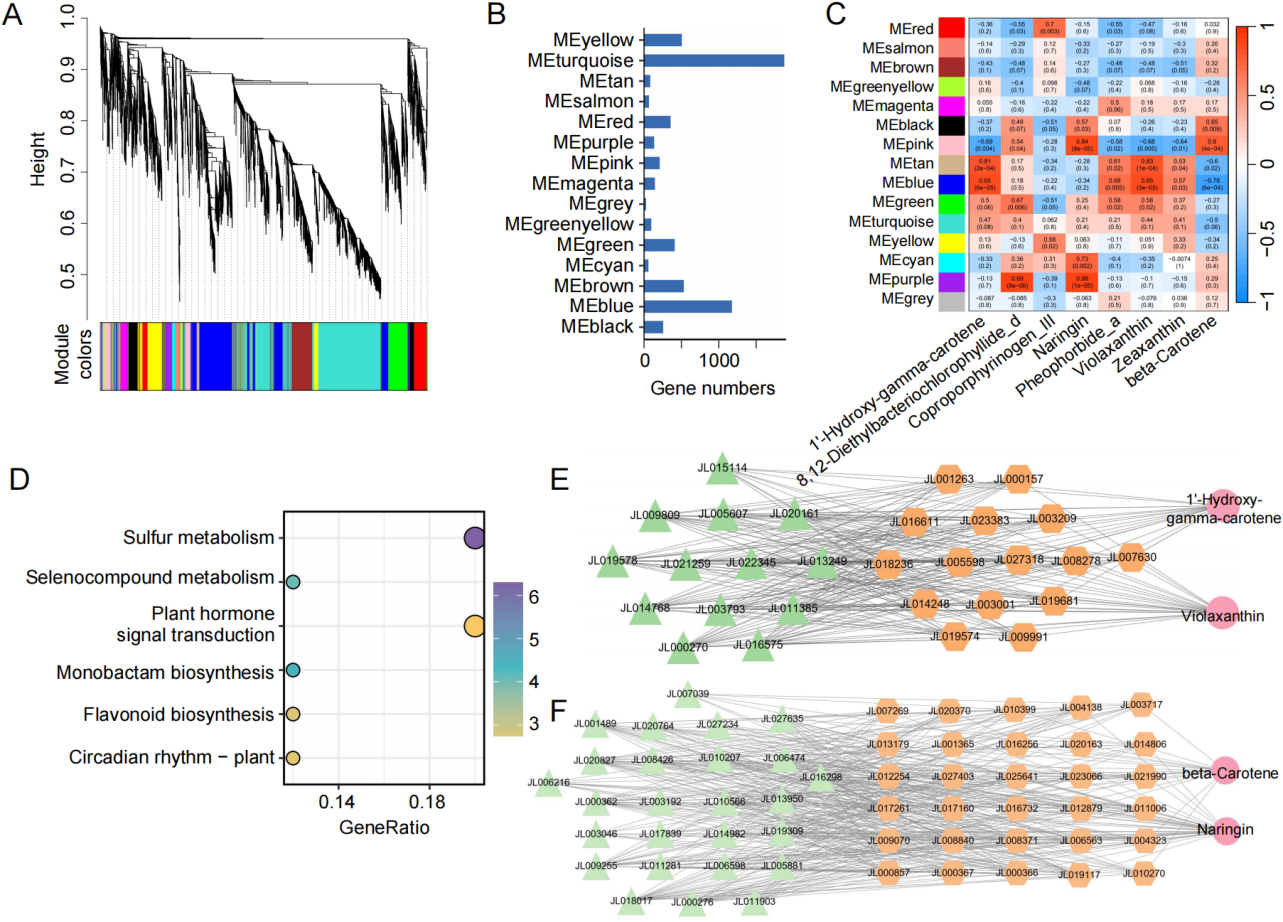
Co-expression gene network analysis associated with *C . ensifolium* pigmentation. **(A)** Hierarchical clustering of modules with different colors identified by WGCNA. **(B)** Bar plot showing the number of genes in each module. **(C)** Heatmap illustrating the correlation between 15 gene modules and 8 pigment-related metabolites. Each row represents a module (indicated by different colors), and each column corresponds to a metabolite. The color scale on the right indicates the module–trait correlation, ranging from -1 (blue) to 1 (red). **(D)** KEGG enrichment analysis of 83 genes in the MEtan module shown as a bubble plot. **(E)** Gene–metabolite regulatory network between genes in the MEtan module and 1’-Hydroxy-γ-carotene and violaxanthin. **(F)** Gene-metabolite regulatory network between genes in the MEpink module and naringin and β-carotene.

To elucidate the genetic regulatory networks underlying pigment-related metabolic differences in *C. ensifolium* leaf color mutants, we performed WGCNA using eight pigment-associated DAMs as phenotypic traits. These DAMs included four carotenoids (zeaxanthin, violaxanthin, β-carotene, and 1’-hydroxy-γ-carotene), three chlorophyll-related compounds (8, 12-diethylbacteriochlorophyllide d, pheophorbide a, and coproporphyrinogen III), and one flavonoid (naringin). WGCNA classified genes with similar expression patterns across samples into 15 distinct modules (**Figure 7A**). Among these, the turquoise, blue, and brown modules were the largest, containing 1, 877, 1, 176, and 532 genes, respectively, while the remaining modules contained between 27 and 505 genes each (**Figure 7B**). Correlation analysis between module eigengenes and metabolite levels revealed that genes in the pink module showed a significant positive correlation with the contents of naringin and β-carotene, whereas genes in the tan and blue modules were significantly positively correlated with 1’-hydroxy-γ-carotene and violaxanthin, respectively (**Figure 7C**). These findings suggest that genes in these modules may influence leaf color by regulating the accumulation of specific pigments.

To explore the biological roles of genes within these key modules, we conducted GO and KEGG enrichment analyses. Genes in the tan module were significantly enriched in GO terms such as sulfate assimilation and adenylyltransferase activity (**Supplementary Table S6**). KEGG analysis further revealed that genes in the tan module were significantly enriched in pathways including sulfur metabolism, flavonoid biosynthesis, and plant hormone signal transduction (**Figure 6D**; **Supplementary Table S7**). Furthermore, we visualized the gene relationships network within modules. For example, the tan module included 15 key genes such as *JL003209*, *JL018236* (encoding BIG, a brefeldin A-inhibited guanine nucleotide-exchange protein), and *JL027318* (encoding GA2ox, gibberellin 2β-dioxygenase) (**Figure 7E**). The pink module included 30 genes such as *JL019117* (KCS, 3-ketoacyl-CoA synthase), *JL000857*, *JL008840*, and *JL006563* (ARF1/2, ADP-ribosylation factor 1/2) (**Figure 6F**). Notably, 13 TFs in the tan module and 26 TFs in the pink module were significantly correlated with both metabolite accumulation and the expression of structural genes in their respective pathways (**Supplementary Table S8**). These results provide candidates for further investigation into the molecular mechanisms underlying leaf color variation in *C. ensifolium*.

## 4 Discussion

The Orchidaceae family is well known for its diverse and vibrant floral characteristics, which have long been the focus of intense scientific investigation. Recently, however, increasing attention has been directed toward the study of foliage variation, particularly leaf color mutations, which also contribute significantly to ornamental value. While earlier studies have utilized cytological, physiological, and molecular biology approaches to explore mechanisms underlying leaf color variation (Qi et al., 2022; Wang et al., 2022), integrative multi-omics analyses remain scarce. In this study, we conducted a comprehensive metabolomic and transcriptomic analysis of *C. ensifolium* leaves with different color phenotypes to uncover the molecular basis of leaf color mutation.

Transcriptome analysis reveals multiple DEGs among various leaf color variants, with the greatest number of DEGs observed in the X vs CK comparison. Notably, 141 DEGs were shared across all comparison groups, suggesting they may serve as core regulators of leaf pigmentation in *C. ensifolium*. These included a variety of TFs, such as *NAC*, *ERF*, *WRKY*, and *MYB* families. Prior research has demonstrated that the MBW complex (MYB-bHLH-WD40) plays a central role in activating flavonoid biosynthetic genes (Baudry et al., 2006; Xu et al., 2014, Xu et al., 2015). Additionally, *WRKY*s have been implicated in the regulation of flavonol biosynthesis in apple, tobacco, and grape by directly modulating *FLS* gene expression (Wang et al., 2018a, Wang et al., 2021; Wei et al., 2023). These findings support our hypothesis that specific TFs may orchestrate pigment-related gene networks, thereby influencing leaf color phenotypes.

Metabolites are the primary cause of leaf color changes in plants. Our metabolomic profiling revealed striking differences in metabolite composition between green and yellow leaves. Four comparison groups (B vs BL, B vs CK, X vs CK, and X vs XL) yielded large sets of DAMs, and 56 metabolites were consistently differentially expressed across all groups. These co-DAMs represent core metabolic shifts associated with leaf color variation and provide a foundation for further investigation into the biochemical changes driving pigment loss or accumulation.

Flavonoids are among the most critical pigment compounds in plants, responsible for a wide range of hues including red, orange, blue, and yellow (Grotewold, 2006). These compounds are synthesized via the phenylpropanoid pathway from phenylalanine precursors (Wang et al., 2018b). In our study, KEGG enrichment analysis of DEGs revealed significant enrichment in pathways including zeatin biosynthesis, phenylpropanoid biosynthesis, and flavonoid biosynthesis. This suggests that flavonoid metabolism is substantially altered in response to leaf color mutations. *CCOAMT* and *TT4* (chalcone synthase) are the key structural genes of flavonoid biosynthesis (Castellarin et al., 2007; Liu et al., 2021a). In *Solanum lycopersicum*, RNAi-mediated suppression of chalcone synthase led to a reduction in total flavonoid levels (Schijlen et al., 2007). In *Syringa oblata*, *SoCHS1* expression was correlated with anthocyanin accumulation during floral development, and its overexpression in tobacco intensified flower pigmentation (Wang et al., 2017). Similarly, in citrus, the deletion of a promoter region in the *CreOMT4* gene cluster reduced polymethoxyflavone levels during domestication (Peng et al., 2024). In our dataset, two *CCOAMT* genes (*JL016896* and *JL016898*) and one *TT4* gene (*JL002997*) showed elevated expression in yellow leaves (B and X types), further linking flavonoid biosynthesis to pigment variation.

Beyond flavonoids, WGCNA revealed that the MEtan module was significantly correlated with two carotenoid-related pigments, 1’-hydroxy-γ-carotene and violaxanthin. Interestingly, genes in this module were also enriched in the flavonoid biosynthesis and plant hormone signal transduction pathways, suggesting a possible interaction between flavonoid and carotenoid metabolism. Carotenoids are vital for photosynthesis, pigmentation, hormone biosynthesis, and stress signaling in plants (Sun et al., 2022). Among the genes in MEtan, several TFs (including members of the *MYB*, *bHLH*, and *bZIP* families) showed strong correlations with carotenoid levels. These results imply that carotenoid-related metabolites may act in concert with flavonoid biosynthetic genes and regulators to shape the leaf color phenotype.

Taken together, our integrative metabolomic and transcriptomic analysis of *C. ensifolium* revealed distinct differences between green and yellow leaves at both the metabolic and transcriptional levels. The identification of shared DAMs and DEGs, especially those involved in flavonoid biosynthesis and key regulatory TFs, highlights the pivotal role of secondary metabolism in leaf color development. These findings not only provide new insights into the molecular basis of leaf color mutation in *C. ensifolium* but also offer valuable targets for breeding programs aimed at enhancing ornamental foliage traits in orchids and other horticultural species.

## Data Availability

The data presented in the study are deposited in the NCBI repository, accession number PRJNA1416868.

## Supplementary material

Supplementary Table 1 Overview of RNA-seq data of *C.ensifolum* and its leaf color mutations.

Supplementary Table 2 KEGG enrichment results of 6716 DEGs.

Supplementary Table 3 –85 TFs in co-DEGs.

Supplementary Table 4 –5252 metabolites identified by metabolomics data.

Supplementary Table 5 Significantly correlated DEG-DAM pairs.

Supplementary Table 6 GO enrichment results of genes in different modules identified by WGCNA.

Supplementary Table 7 KEGG enrichment results of genes in different modules identified by WGCNA.

Supplementary Table 8 Transcription factors in the gene-metabolite regulatory network of MEpink and Metan.

Supplementary Figure 1 The soft threshold power (β) is calculated using the pickSoftThreshold function of WGCNA.

Supplementary Figure 2 The volcano plot shows the results of differential expression analysis of the four comparison groups (B vs BL, B vs CK, X vs CK, and X vs XL).

Supplementary Figure 3 Comparison of qRT-PCR and fpkm for five differentially expressed TFs (JL003847, JL014616, JL015258, JL022919, and JL024060). Bars represent fpkm, and circles represent the relative expression levels in qRT-PCR experiments.

Supplementary Figure 4 Regulatory Network of co-DEGs and co-DAMs.
