# Supplementary figures and images for: Integrative transcriptomic and metabolomic analysis reveals the molecular basis of leaf variegation in Cymbidium ensifolium
Source: Front Plant Sci. 2026 Feb 12;17:1712811. doi: 10.3389/fpls.2026.1712811 (PMC12935976; doi:10.3389/fpls.2026.1712811)

### Scale independence

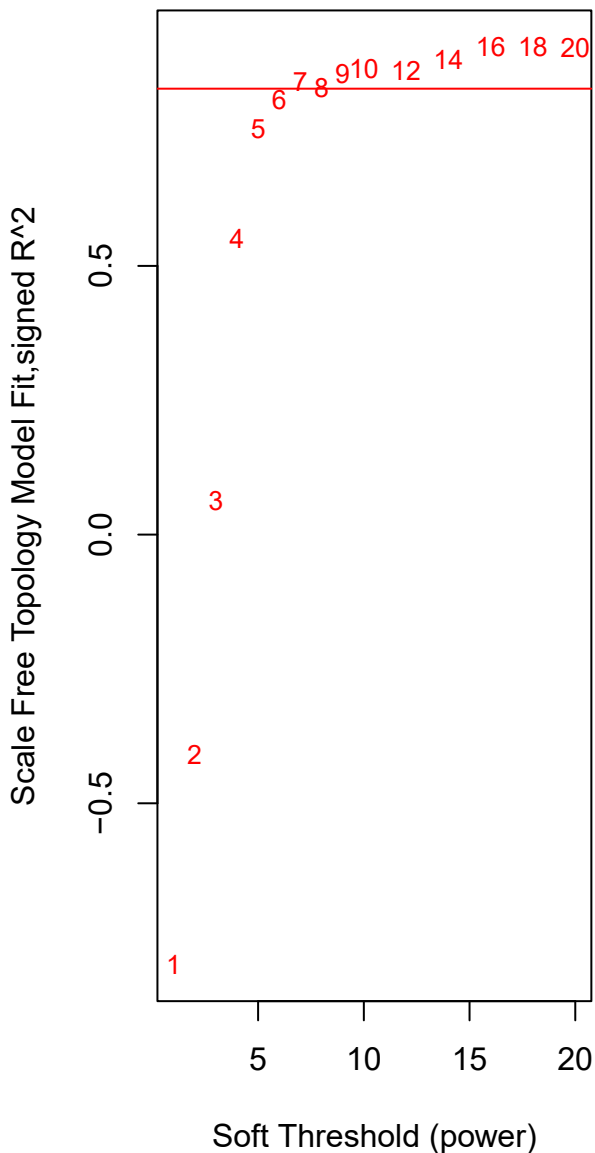

### Mean connectivity

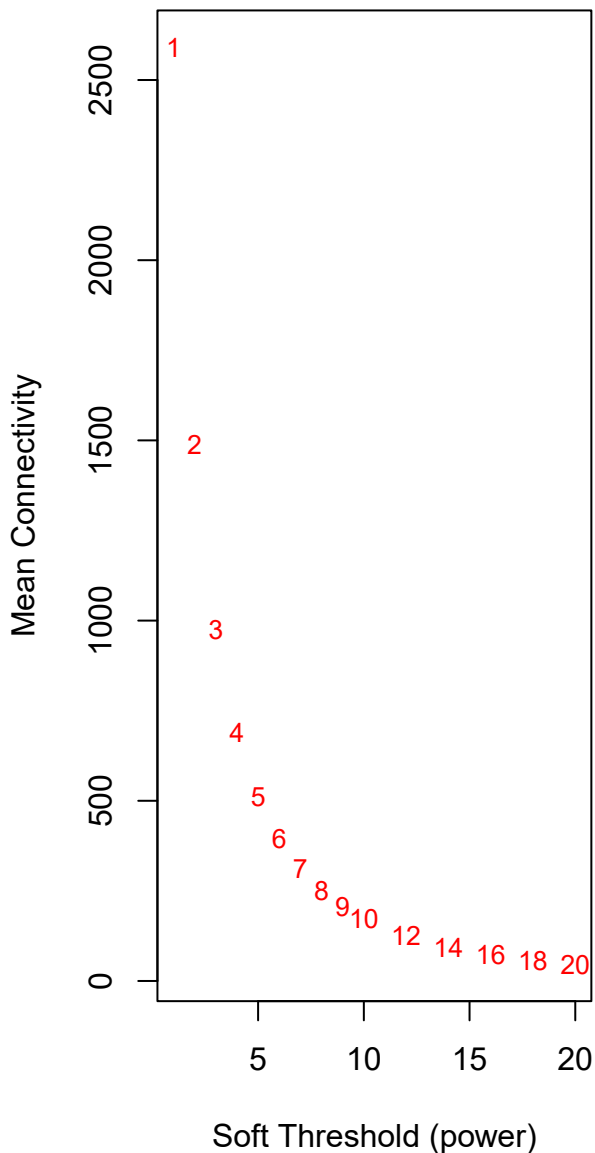

Supplement: Supplementary Figure 1 — The soft threshold power (β) is calculated using the pickSoftThreshold function of WGCNA. [file DataSheet1.pdf]

BvsBL

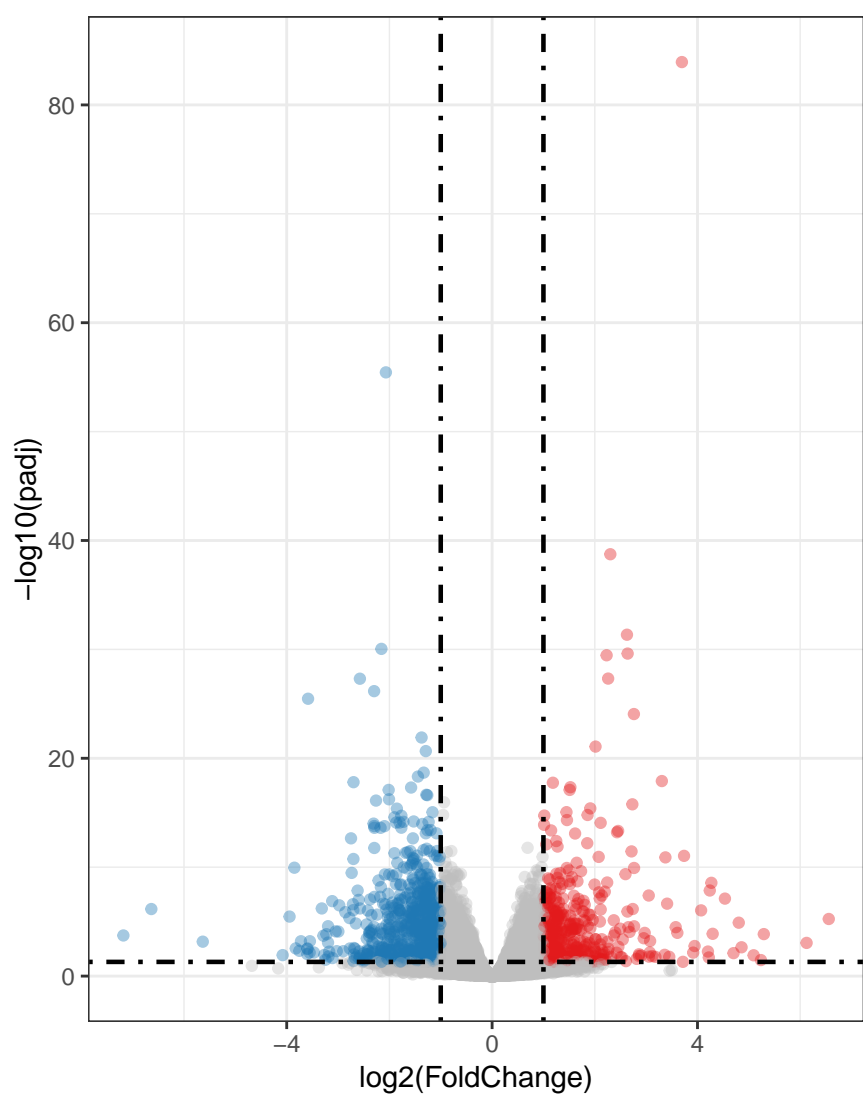

BvsCK

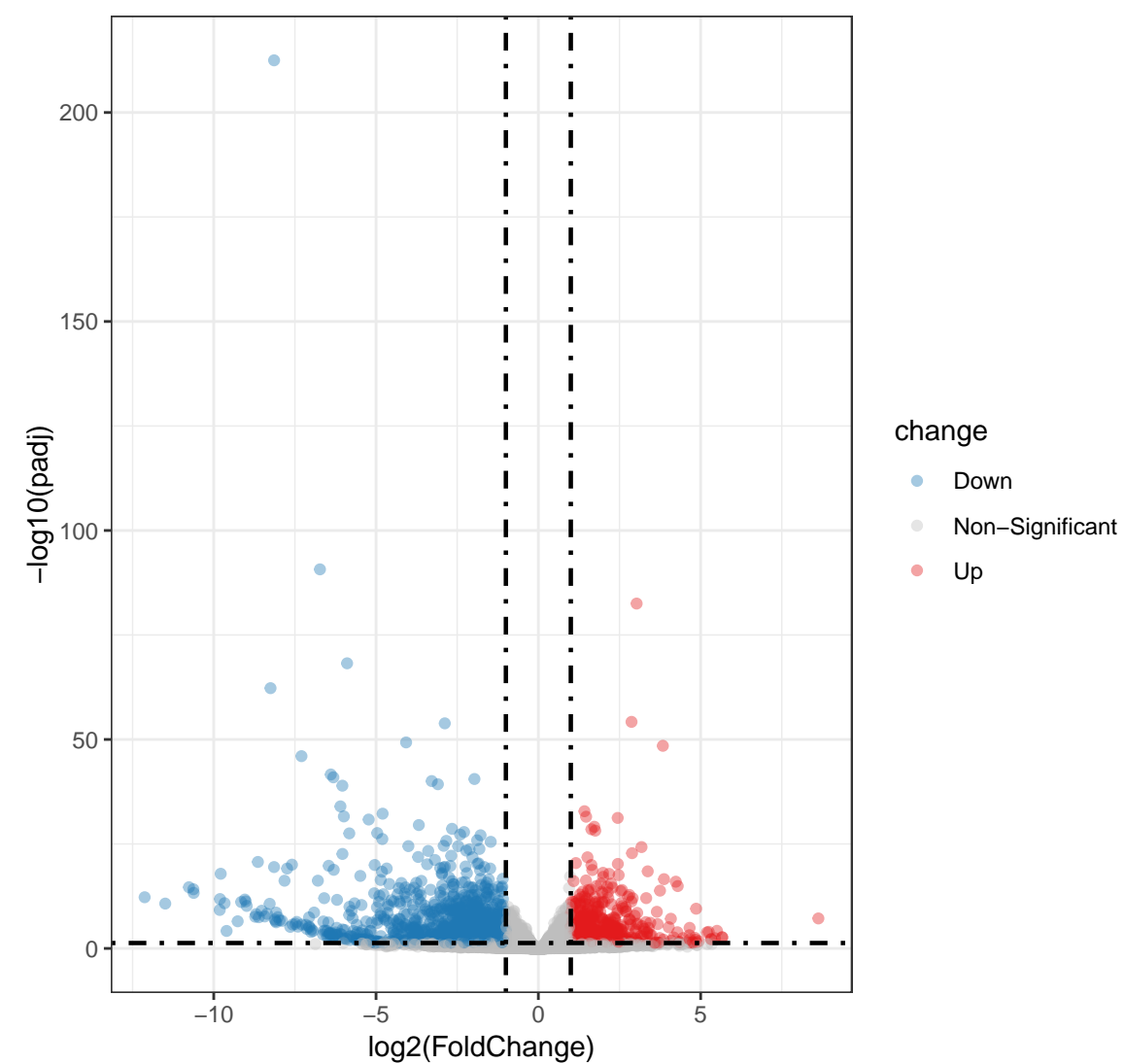

XvsCK

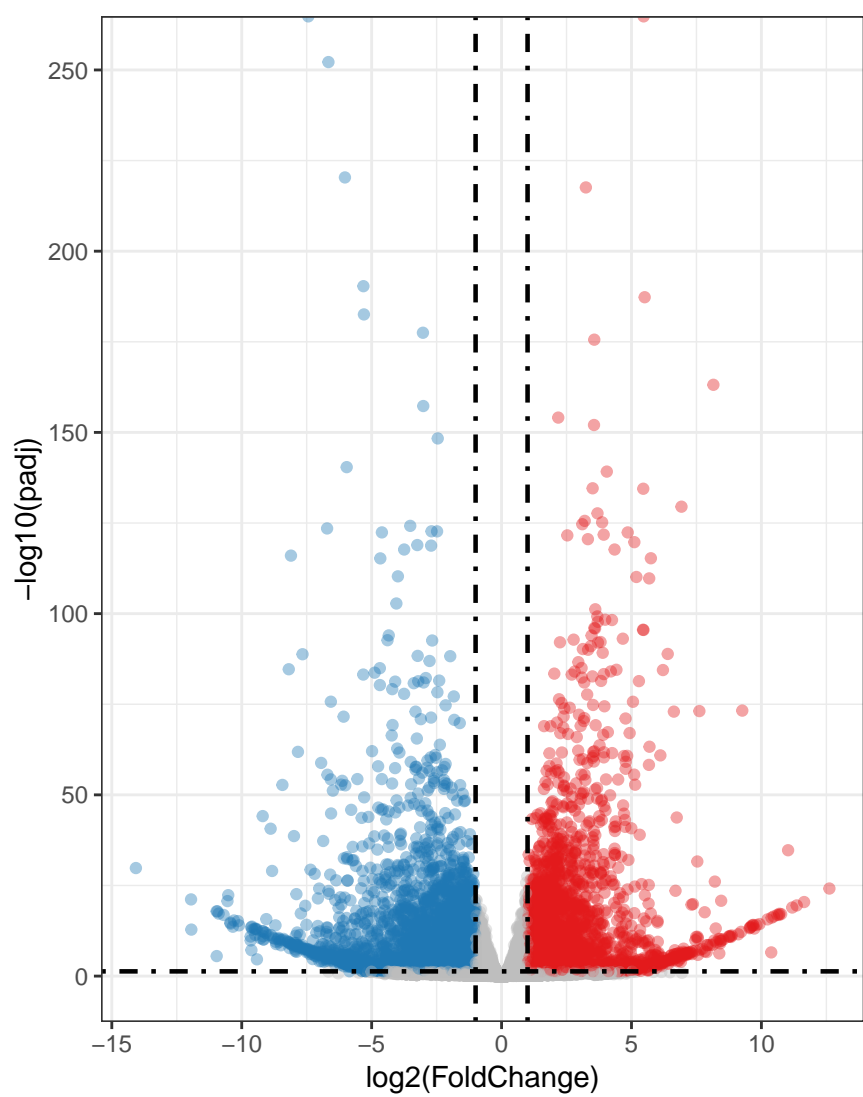

XvsXL

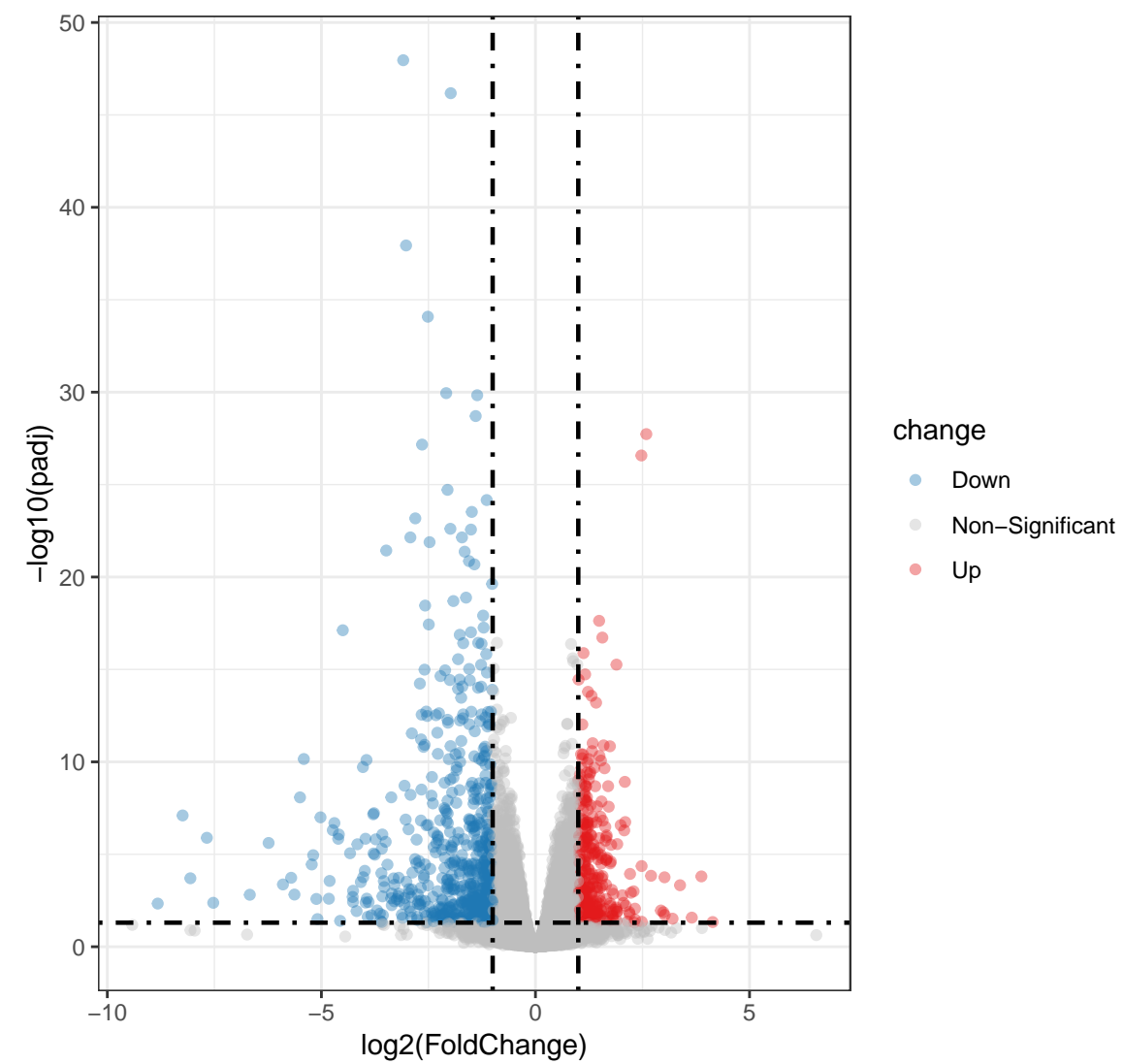

Supplement: Supplementary Figure 2 — The volcano plot shows the results of differential expression analysis of the four comparison groups (B vs BL, B vs CK, X vs CK, and X vs XL). [file DataSheet2.pdf]

JL003847

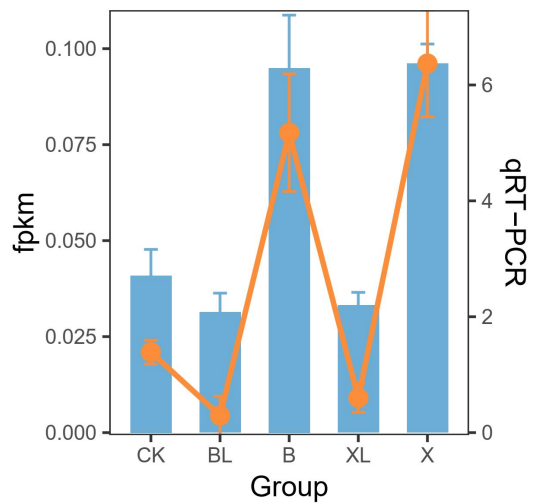

JL014616

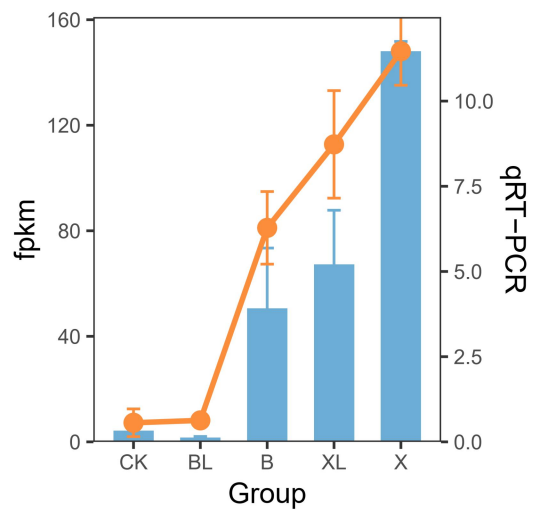

JL015258

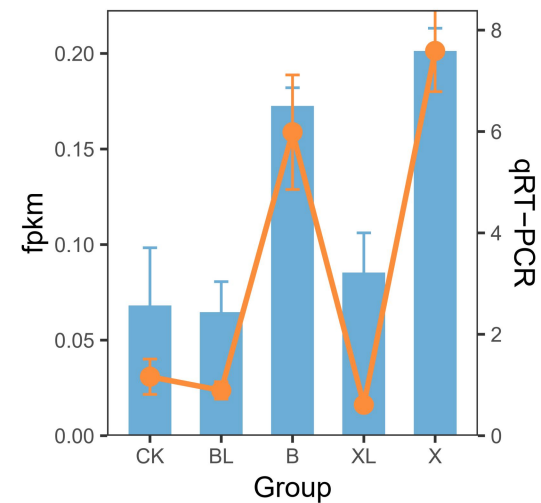

JL022919

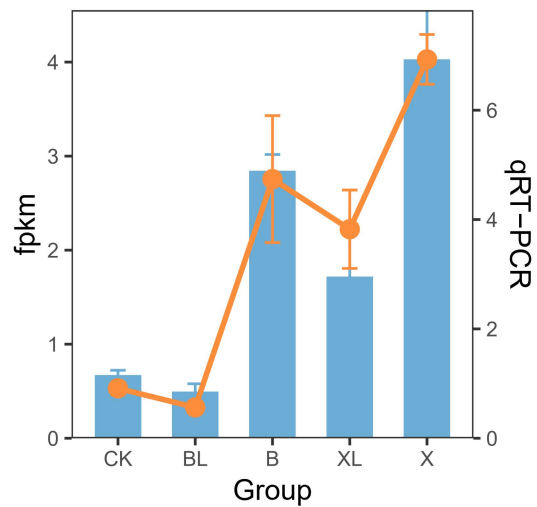

JL024060

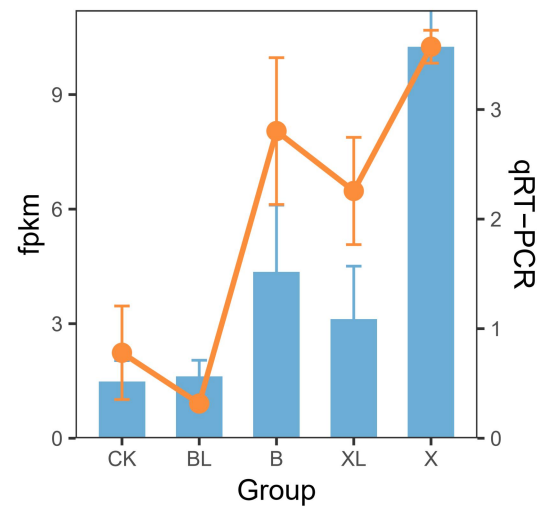

Supplement: Supplementary Figure 3 — Comparison of qRT-PCR and fpkm for five differentially expressed TFs (JL003847, JL014616, JL015258, JL022919, and JL024060). Bars represent fpkm, and circles represent the relative expression levels in qRT-PCR experiments. [file DataSheet3.pdf]

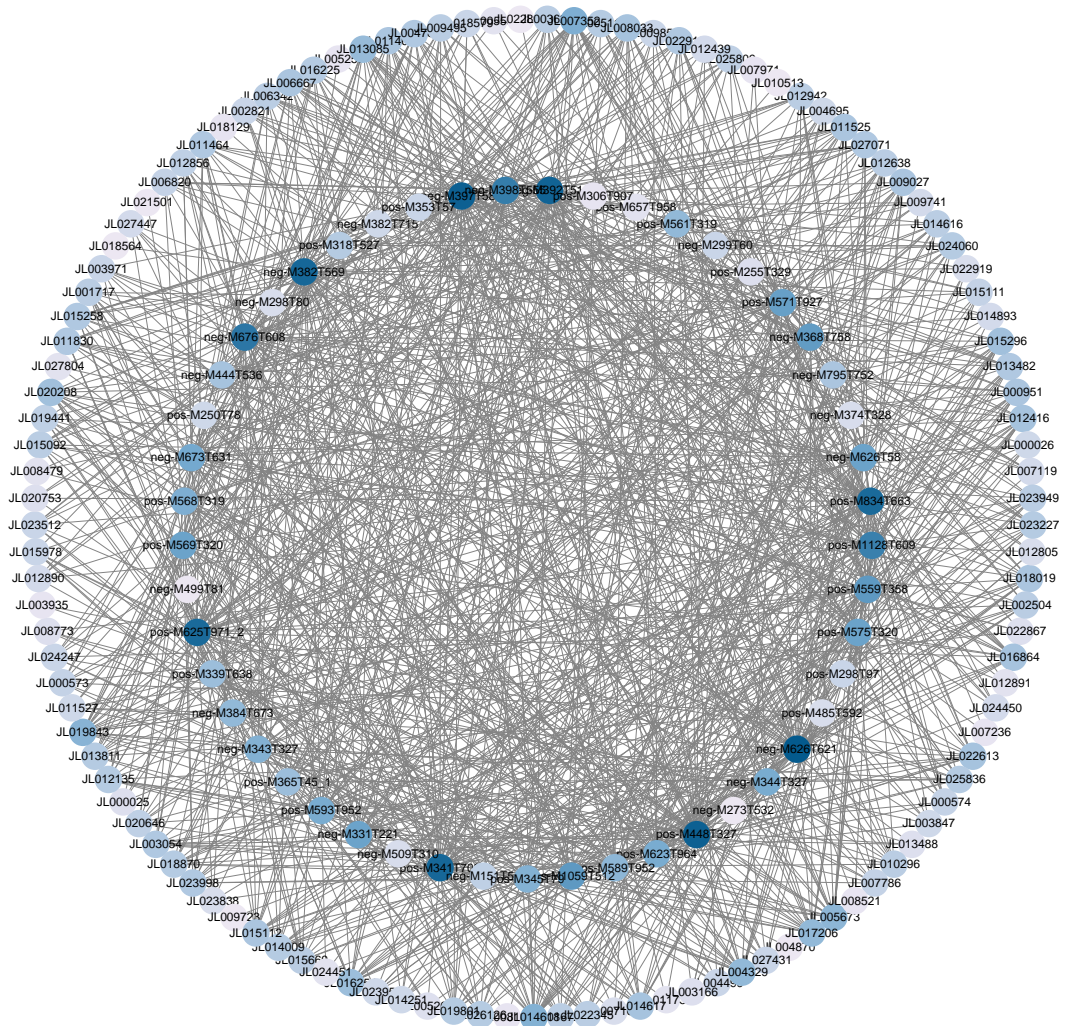

Supplement: Supplementary Figure 4 — Regulatory Network of co-DEGs and co-DAMs. [file DataSheet4.pdf]
