# Supplementary material for: Integrative transcriptomic and metabolomic analysis reveals the molecular basis of leaf variegation in Cymbidium ensifolium
Source: Front Plant Sci. 2026 Feb 12;17:1712811. doi: 10.3389/fpls.2026.1712811 (PMC12935976; doi:10.3389/fpls.2026.1712811)
Supplement: Supplementary file 13 [file DataSheet5.pdf]

>JL003847ATGGGCGACGGCGAAGATGTGAAGAAGGTTGGGGTGAAGG
AGGAGAAGGTGGCGTTTTACAAGTTGTTCTCCTTCGCGGACTCCAAGGACG
TGGTTTTGATGTTGGTGGGCTTCATTGCCGCGGTAGCCTCTGGGCTTGCAAT
GCCGCTCATGAGCTTCATCTTCGGCAAGCTTAGCAATGCCTTCGGCGTCGC
CAACCGGGATAATGTTGTTCATGATGTTTCTAAGGTTGTCATACAATTTGTA
TATTTAGCTATTGGTGCTGGTGTTGCATCATTTCTACAGGTGTCCTGTTGGAT
TGTGACCGGAGAAAGGCAAGCTGCACGTATACGTAGTTTGTACCTAAAGA
CCATTTTAAGACAGGATATAGCATTTTTTGATAAGGAAACAAACACTGGTG
AAGTGATAGGGAGGATGTCCGGTGACACTATACTTATTCAAGATGCCATGG
GTGAGAAGGTTGGGAAGTTCATACAACTTACTTCAACATTCATTGGGGGCT
TTATCATTGCACTTATCAAAGGATGGCTTTTGACGCTGGTCATGATTTGTTG
TATTCCTTTCATTGTCACGGCTGGTGCAACAATGTCTTTAATAATATCAAAA
GTGTCAACTCGTGGACAATCGGCATACGCCGAAGCGGGGTCTGTTGTTGA
CCAAACTGTTGGCTCCATTAGGACAATTGTGTCATTCTCAGGCGAGAAGCA
AGCCATCCAGAAGTATAATAAATTCATTAATAATGCCTACAAATCCATTGTT
CATCAAGGAACTGTTGCTGGACTTGGTATTGGCTTCGTTATTATGTTTGTATT
TTGCAGCTATGCCTTAGCTGTCTGGTATGGTTCTACGCTGATCATAAATAAA
GGCTACAGTGGAGGGGAAGTCATCAATGTTATGCTCGCTGTTATGATAGGT
TCAATATCCTTGGGCCAGGCATCCCCCAGTTTAAGTGCATTTGCAGCAGGG
CAAGCTGCTGCATACAAGATGTTTGAGACAATTCATCGGAGACCAGAGATT
GATTCTTATGACAACAAGGGCGTTGTCTTAGAAAACCTCAAGGGCGACATA
GAACTGAAGGATGTTTACTTCAGCTACCCAGCTAGACCTGATCATTTGATAT
TTAATGGCTTTTCTTTGCGTGTGCCACGTGGTACAACTATGGCCCTAGTAGG
TGAAAGTGGTAGTGGGAAGTCAACAGTGGTTAGTCTGGTTGAGAGGTTTTA
TGATCCTCAAGCTGGTGAAGTATTGATTGATGGCATCAACCTGAAGAATCT
CAAACTAAGGTGGATACGGGAACAGATTGGCCTCGTTAGCCAGGAACCCA
TCCTTTTCACAACCACCATCAAGGAAAACATTTCTTATGGAAAGGAAGGTG
CTAGTGCTGAAGAAATTAGGATAGCAATTGAACATGCTAATGCTTCAAAGT
TCATATACAAGTTGCCTAATGGCCTCGAGACAATGGTTGGGGAGCATGGTA
CTCAGCTATCTGGTGGACAAAAGCAGAGAATTGCAATTGCAAGAGCAATTT
TAAAGAATCCTAAGATCCTGCTTTTAGATGAGGCAACCAGTGCATTGGATG
CAGAGTCCGAGCGAGTTGTTCAAGATGCTCTCATAAATATTATGGTAGACC
GAACAACCATAATTGTTGCTCATCGCTTGATTACCGTGAAGCATGCTGATA
CCATATCAGTTGTTCAGCGAGGAAAGCTTGTTGAACAAGGTTCACACTCAG
AATTGATTGAGAATCCAGATGGAGCTTACTCCCAGCTTGTGCGACTGCAGG
AACTAAACAAAGAATCAGAATCAGCACCTTCAGAGAGTTTAGCTGAAAGA
GGTCTAAGCAATGGTGCTTCTATCTCTTTTAAAAAATCAGAATTTCTTGCTTT
TTCCCAAACGAGATCGATCTCTCGCGGCTCTTCAAGTGGAGGAAGCAGAA
GGCTTTCATTTACACAATCTGTTGGGTTGCCCGGTGTGATAGAAGCTGAATT
AAACGGCCCAGAAGCATACGATGAATGGAAGAGAGATCGTAGCAATACTA
AGGAAAGGAAAGAAGTTTCTATAATGCGGCTTGTTTACCTCAACAAGCCAG
AGATCCCAGTTCTTCTTGTTGGATCCATTGCTGCAGCTGTCCACGGAGTCAT
CTTTCCTGTCTATGGAATCTTGATTTCTAGTGCCATCAAGACATTCTATGAA
CCACCACATCAACTCCGAAAGGATTCCAGGTTTTGGGCCCTGATGTATGTA
CTATTAGGTTTCATTTCTTTCCTTGCTGTGCCAGTACAATATTACTTGTTTGG
AGTTGCTGGTGGGAAACTCATAGAACGCATCCGTTCACTGTCATTTGAGAA
GGTAGTGCACCAGGAGATCAGCTGGTTTGATGAACCTGCAAATACGAGTG
GTGCAATAGGTGCACGGCTGTCTACAGACGCATCGTCTGTGAAAAGCCTA
GTTGGTGACACTTTAGCTTTGATTGTTCAGAACTTAGCAACGGTTACAGCAG
GGGTAGTTATAGCCTTCGTTGCAAACTGGAAGCTTGCCCTTATGATCATGG
TAATTGTACCTCTGGTTGGACTGCAGGGTTATGCGCAGATGAAATTCCTTCA
GGGATTTAGTGCTGATGCTAAGATTAAATATGAAGAAGCCAGTCAAGTGGC
AAGCGATGCAGTCTCTAGCATCCGCACAGTGGCTTCCTTTTGTGCAGAGGG
GAAGGTGATGGATGCTTATCAAAAGAAATGTGAAAATCCGGTGACGCTGG
GAATTCGGCAGGGAGTAATAAGTGGACTTGGTTATGGCTTTTCATTTTTCAT
CATGTATTGTACCTTTGCTCTGTGTTTCTACGTTGGAGCTCGTTTTGTACATA
ATGGAAGTGCAGCTTTTACCGATGTTTTCAGGGTGTTCTTTGCTTTGACCAT
GGCAGCTAATTCAGTTTCACAATCGAGTGCACTCGGTCCAGATGCTACAAA

AGCCAAAGACTCAGCTGCCTCTGTATTTGGAATTCTTGATCGGAAATCCAA
GATTGATGCAAGTATTGATGAGGGGTTGATGCTGGCAGAAGTTAAGGGGG
AGATTGAATTCCAGCATGTCAGCTTCAGGTACCCAACACGTCCAGAAGTGC
AGATCTTCACTGATCTGTGTTTGAGAATGCCATCTGGAAAGACAGTTGCGC
TAGTTGGTGAGAGTGGCAGCGGAAAATCTACAGTGATCGCTCTCATTGAGC
GATTCTATGATCCTGATTCAGGTGCCATTGTCATTGATGGGGTTGAGATACG
CAAGTTCAAACTCAGCTGGCTTAGGCAGCAAATGGGATTAGTAAGCCAAG
AACCTGTGATGTTGAATGGTACAATACGCTCCAATATAGCCTATGGCAAGC
AAGGTGATGTGTCTGAGGATGAGATCATTGCCGCCGCCGAGGCAGCTAAC
GGGCACCGCTTCATTTCATCGCTTCCTCAGGGCTATGATACGAATGTCGGA
GAGCGAGGTGTTCAGCTCTCAGGCGGGCAGAAGCAGCGCATCGCCATTGC
AAGGGCTATGATCAGGAACCCAAAGATTCTTCTACTTGACGAGGCCACCA
GCGCCCTGGATGCGGAATCGGAACGTGTGGTGCAGGAAGCGCTCGACCGT
GTGATGGTGGGCAGGACGACGATCAGCATTGCTCATCGTTTATCCACAATT
CAGGGCGCCGATGTTATTGCTGTGGTCAAGAATGGTATTATTGCCGAGCAA
GGGCGGCATGAGACGCTTATGGGCATGCCGAACGGTGCTTATGCTTCACT
AGTCACCCTTCATACGTCATCCTGA>JL024060ATGGCACCAGAACCCA
CTATTGAAGAGGACAATCACCCCACAACCAGCTTCCCACAATCTCTCTCCG
ACCTCGACCTCAGTTTCACAAGCAGCTCCGCCTCCACTTCCTCCTTCGCCAC
CACATTCGGCGGAGGCCCATCTAGTGCCCGCAGTAGCCTCAGCCTCCCCTC
CTCTTCCTCCTCTTCCTCCTCCCTCAAACCTCTCCCCCACCGCTGCCTCGACC
CCCACTGGTCCGCCCTTCGCGCTGCCGCGAACCTCTCTCCCGACGCCTCCA
TCCACCTCCACCACCTCAAACTCCTCCGCCACATCGGCTCCGGAAACCTCG
CCCGCGTCTTCCACTGCCGCCTACACGGCTTCGACAACCTCGACTTTGCTCT
CAAAGTCGTCGACCTCGACGCCTCCGTCAGTACTCTCTCTCACGTACGAGC
CGAGGCCCGCGTTCTTGCTTGTCTCGACCACCCCTTTCTCCCCACCCTCTAC
GCCCGACTCGACGCCTCGCACTACGCCTGCTTCCTCATTGACTACTGCCCC
GGCGGCGATCTCCACTCCCTCCTCCGCCACCGACCTAGACATCGCCTTCCA
CTTCCCGCCGTCAAATTCTACGCCGCTGAGGTCCTCCTCGCACTCGAGTAC
CTCCACGCCCTCGGTTTCGTTTACCGTGACCTGAAGCCTGAGAACGTCCTC
CTTCGCACCGACGGCCACGTCATGCTTTCTGACTTCGACCTCTGTTTCCACT
CCGATGTCTCCCCTGTTCTCCACCGCCGGAATCGCGGGAGATCAGTGGTAG
AGCTGGAGTTCGTGGCGGAACCAGAATCGGCCTTCTCCCGTGCATGCGTG
GGGACGCACGAGTACCTCCCCCCGGAGATCGTGGGCGGAGCCGGCCACG
GTAACGGAGTTGACTGGTGGGCATTTGGGGTTTTCATCTATGAGCTTATCTA
TGGTCGGACGCCGTTCGAAGGTGGGAGCAAAGAAGCAACTTTGAAAAACA
TTCTCTCAAGGGAGGTGAGATTTCCCGACGGCGAGGCCGACCAAAGGCGT
GCGGCAGTGGCTGCGAGGGATTTGATTTCGCGACTGCTGGAGCGCGATCC
ACGACGGAGGATGGGGAGCGTGAAGGGAGCGACAGAGATCAAGCAACAT
CCATTCTTTGATGGTGTAAGCTGGGCGCTTATTCGTATGATGAAGCCACCA
GTGGTGGTGGGTCACGCCGGCCCCACCGCGAAGAAGCAAGTTTCGAGGG
AGGGGAAGCGATGGTGGACGTGGAAGTGGAGCAGTTGTAACAGTAAAGG
AAATGAGAAAAGTGTTGCTTACTGGAAGAAGATTAGGGAAATGAAAACGA
AACAGTGA>JL014616ATGAAATGCGAAGCTGATGAATGTGGCTCAAA
CACGAAGGGCCTCCACTTTCTTCTCGTCCCTCTCATGGCACAGGGCCACAT
GATCCCCATGGTCGACCTAGCTAGGTTCCTCGCGGCCCGTGGCAGTCTGGT
CACCTTTGCCACTACTCCGGTCAACTTGGCTCGCATTCAACCCATCATCGAT
CGGGCCGTTATCTCCGTCCTTCCGATCCGTTTCCTAGAGCTCCGCTTTCCGG
TTACTGACATCGGCCTCCCTGATGGCTGTGAGAACGCCGATCTCATCCCCT
CCGCAGATCTCTTCTTCCCATTCATGAATGGTCTATCCCTTCTACGTGAATC
ACTCGATGCCCACTTGCGCATCCCCGACCCGGCTCAGTGGCCTAAACCCGC
CTGCCTCATCTCTGACAACCTTCAACATTGGACAGCTGATGTTGCCCGCAC
CCTTAACATCCCCCGTCTCATCTTTCATGGCCCATCTTGCTTCTTCCTTTTCTA
TGACCTCTTCATCAACCAACGTAGGGCCGAGCTTGAGGCTGCAGTTGAGGC
AGCATCGGATGGGTCCATCATGCTTCCGGGCTTCCCACATTCCATTCGACTC
TACAAGCACGAGGTGATATCTGTCTTGCGCTCGAACGTTGATTGGTCCAAA
TACCTGGACTCTGTCAGGGTGGCAGAAGAATCTGCCGACGGCGTAGTTGT
CAATAGTTTCGCTGAGCTCGAGCCTTGGTACTTCGAGAAGTATCAGGAGGC

GACGGGCAAGCCAGTGTGGCCTGTTGGACCGCTCTCACTCTATGAAGAAG
AGCTCGATGTCAAGGCGGCACGCGGACGAGTTTCATCGATCGACAACGCG
CTCCTCTTCCGGTGGTTAGACAGGCAAGAGGCCGGGTCAGTGGTGCTCGTC
AGCTTCGGAAGCATGGTACGCAACACAATGGCCCAGCTCGTGGAGTTGGG
CCACGGGCTAGAGGCTTCGGGTCGACCGTTTGTTTGGGTGCTGAAGGAAG
CAGCGGAGGGGAAGATCCCAGGGGTGGAGGAGTGGATAGCAGAGTTTGA
AAGAAGGGTGAAAGGGAGGGGAACGGTGATAAGAGGGTGGGCGCCACA
AACACTGATATTGGGGCATGTAGCGGTGGGGGGATTCGTAACCCATTGCG
GGTGGAATTCGACGCTGGAAGCCCTGGTGGCGGGGGTGGCGATGGCGAC
GTGGCCGCACTTCTACGACCAATTTTTTAACGAGCGATTGGTGGTCGATGT
GCTCAAAATCGGCGTGTCAATTGGCATCAAGGAGTTGAGTTCATTCGCTGT
AGAGGATGATAAAGATGCGGCGGTGAAAGTGAAGAGTGAGGAGGTTGAG
AAGGTGTTGGAGAAGTTGATGGGGGGAGGAGAGGAAGGGGAAGAGAGG
AGAGAGAGGGCGAGGGAGCTCAGCGAGAAGGCGAGGATGGCTATGGAG
AAAGGAGGGTCTTCCTGGAAGGGATTACAAGATGCAATCAACTATGTGTTA
GAGAACCAGAAGAAGTAG>JL022919ATGTGGGTCGGCGCGCGGAAG
AGGAGCGATCCGGGTCGCACGGGTGGCGGGCTGGTCGTGTCGCGAGACA
CGTGGCGAATGGCGAGCTGTAGGTGGAAGCAGGCTGGCCACGCGCGCAC
GCGCGGACAACGCGTGCGCGGGGCGAGTGGGCGCTGCACAGCAGTCAGC
GGTGGCCTCACCATCCGGTGCCAGGCGAAGGATAAACCCCAGGATGTTGA
GCCCGGATCCACCGTCAACCCTGCCGAAGGGAACCAGACGGGTTTCACGC
GTGTGCAGGCAAAGAGCACAACCATCTGGGACGCGCTGGCCTTTAGTGGG
CCGGGTCCTGAGCGGATCAACGGCCGGCTTGCTATGGTCGGGTTCGTCTCG
GCGCTGGCGGTCGAGGCAGTGCGGGGAGAGGATTTGGTGGCTCAGCTCG
CCGATGGCGGCGTGTTCTGGTTCATCGGAGCGGCGGCGCTGTTCTCGGTG
GCGTCGCTGGTTCCGCTATTTCGCGGAGTCGACGCGCCGGAGAGGTCGAG
TGGGTTCATGACCGCTGACGCGGAGCTATTGAACGGCCGGTTTGCTATGCT
GGGTCTGGTCGGGCTCGCCTTCACTGAGTTTCTAAAGGGTGGGCCACTTGT
TTAG>JL015258ATGCTACACTTTTCTTTTACCTCTCTCTTCCCCTTCTTCT
TCTTCTTCTTCTTCTTCTTGCGCTCTTTATCCGCTTCTCCCTTAGCTTACTCGC
GCCTTCTCCCGATCATCAACGACCAGATTCAAACCTACATTGTCCATGTCAA
GAAACCCCAGCTTCTCTTCTATTCTCCCTCTGATCTCAAGAGGTGGCATACT
TCCTTCCTCCCCAACACCACCCTCTCCTCCGGCGAACCCCGCCTGATCTATT
CCTACGGTGACTCCGTCACGGGTTTCGCCGCTCGCCTTACCCCTCAAGAAG
CTAATGCTATGGAATCCATCGACGGCTTCCTGTTCGCCCACCCTGATCGCC
CCCTCTTCCCCCATACCACATACACTCCCCAATTCCTTAACCTCGATGATTG
GACTGCCAGCCGCGGAAGCGTATGGGAGAAGTCCTTCTATGGGGAGGGC
GTTGTCATCGGCGTGATCGACACCGGCATTTCTCCAACCCACCCTTCTTTCA
ATGAAGAAGGAGTTTCGCCCCCACCGATCAAATGGAAGGGCAAATGCTCC
ATCGCCGCTTGCAACAACAAACTCATTGGCGCCAATGCATTTCGTGGCGGC
GATCGGAAGGCTTCTCCGATCGATTTCGATGGGCACGGAACACATGTCAC
GGGAATCGCTGCAGGGAACTTTGTAAGCCATGCCGATGTCGGTGGAATGG
TGCACGGAAGGGCCTCGGGGATAGCCCCCAAAGCTCACGTTGCTATCTAC
AAAGCCTGCTTCGAAGACCAATGTAGGGATAGCGATGTATTGGCTGCTGTC
GATCAAGCGATTCATGATCGAGTCGATGTCTTGTTGATCCCTCTCGGTGGG
ATGCCCCTGCCTTTGTATCAGGACAGCGTCGCAGTCGCCTCTTTTGCCGCG
GTTGGGAAGGGGATCGTTGTTTGCACCTCGGTTGGAAATGGCGGACCGAG
GACTGGAGAGATCAGCCATGACGCTCCATGGTTGGTGACCGTCGGCGCGA
GTACCCTCGATAGAAGAGTTAGAGCAGTGGTGAGGCTTGGGAATGGAGTG
GATCTGATGGGGGAAAGCGGGTTCCAGCCGCCAAGCGATGAGTTCCCTCC
TATCTTCCTGCCCATAGTTTTCCCGGGCATAGACGGCGACTCAGGCGCTGC
GAATTGTCACAATCGTTCCCTGGATGATCTCGATCTTAGGGGAAAGATCGT
CCTCTGCTTTGCCGGCGACGAGGTTAACACCGACAAGACCCGCGCTGCGA
TGAACGCAGGCGCTGCCGCCGTGATATTGATGAACCGGCGGAGCCACGGC
TTCACCACGCGCTCCGAGATGCATGAGCTGCCGGCGTCACATCTAAGTTAT
TTGGACGGGCAGCGTCTGCGAAGCTACGTCTACTCTACGTCGTCGCCGGTG
GCGGCGATTCTATTCAACAGAACGATATTCGGGGCACGGCCGTCGCCAGC
GGTCGCCGCGTTCTCCTCGCGAGGGCCTAGCTTGATGAACGGTGGTGTGTT

GAAGCCAGACATCGTCGCCCCAGGCGTGAACGTTCTCTCCGCGTGGCCGC
CGGTGGCCACCTCGCCTCTGTTTGCTTTCATGAGCGGGACATCCATGGCCG
CGGCTCACGTCGCCGGCGTCGCTGCTCTAGTAAGGAGAAGGCACCCGGGG
TGGTCGCCGGCAATGATTCACTCAGCTCTTATAACATCAGCAACAAACCAG
GATCTCGACGGGCATCAGATGTTCGATGAATTGTCATACAACAGCACGGC
GAGCCTCTTTGCCGCCGGGGCTGGGCAGCTGCACCCGCAGGGAGCACTTA
ATCCCGGTCTGGTGTATGATATCGAGCAAAAGCATTACTTGCGCTATCTAT
GTGGATTGGGTTATACCAACACGCAGATGTGGGCTTTCGCGAAGCAGTAC
GTTTCATGCAACAAGAGCATGGAGCTGGCGGAGTTGAACTACCCATCAATC
TCAGTGAGGTTGGGATCGAGCTCGGAGAAAAGTGTGGTTCGAACTCTGAA
GAGCGTGAGGAAGAGGAGCACGGTGTACTGGGCGAGGATTGAGGAGCCG
AGAGGAGTGAGAATAGACCTATCACGTTATGAGCTGAGCTTCTCAAGGGT
GAACCAGGAAGAGAGTTTTGAGATAAAATTTAGGATCCAAGGTCGCAGAC
CAAGAAAAGGCCACGTTTCGCAAGGGAGGCTTTCCTGGGTTTCAAGAACG
CGTGTGGTGAGTAGCCCCATCTTAGTTACTTTTTTATGA
